# Anticancer potential of alkaloids: a key emphasis to colchicine, vinblastine, vincristine, vindesine, vinorelbine and vincamine

**DOI:** 10.1186/s12935-022-02624-9

**Published:** 2022-06-02

**Authors:** Praveen Dhyani, Cristina Quispe, Eshita Sharma, Amit Bahukhandi, Priyanka Sati, Dharam Chand Attri, Agnieszka Szopa, Javad Sharifi-Rad, Anca Oana Docea, Ileana Mardare, Daniela Calina, William C. Cho

**Affiliations:** 1 Department of Biotechnology, Kumaun University, Bhimtal, Uttarakhand 263 136 India; 2 Facultad de Ciencias de la Salud, Universidad Arturo Prat, Avda. Arturo Prat 2120, 1110939 Iquique, Chile; 3 Department of Molecular Biology and Biochemistry, Guru Nanak Dev University, Amritsar, Punjab 143 005 India; 4 G.B. Pant National Institute of Himalayan Environment, Kosi-Katarmal, Almora, Uttarakhand 263 643 India; 5 Graphic Era University, Dehradun, Uttarakhand 248 001 India; 6 Chair and Department of Pharmaceutical Botany, Medical College, Jagiellonian University, Medyczna 9, 30-688 Kraków, Poland; 7 Facultad de Medicina, Universidad del Azuay, Cuenca, Ecuador; 8 Department of Toxicology, University of Medicine and Pharmacy of Craiova, 200349 Craiova, Romania; 9 Department of Public Health and Management, Carol Davila University of Medicine and Pharmacy Bucharest, 050463 Bucharest, Romania; 10 Department of Clinical Pharmacy, University of Medicine and Pharmacy of Craiova, 200349 Craiova, Romania; 11 Department of Clinical Oncology, Queen Elizabeth Hospital, Kowloon, Hong Kong People’s Republic of China

**Keywords:** Alkaloids, *Vinca*, *Catharanthus*, *Colchicum*, Anticancer, Microtubule-targeting agents, Antimitotic, Apoptosis

## Abstract

Cancer, one of the leading illnesses, accounts for about 10 million deaths worldwide. The treatment of cancer includes surgery, chemotherapy, radiation therapy, and drug therapy, along with others, which not only put a tremendous economic effect on patients but also develop drug resistance in patients with time. A significant number of cancer cases can be prevented/treated by implementing evidence-based preventive strategies. Plant-based drugs have evolved as promising preventive chemo options both in developing and developed nations. The secondary plant metabolites such as alkaloids have proven efficacy and acceptability for cancer treatment. Apropos, this review deals with a spectrum of promising alkaloids such as colchicine, vinblastine, vincristine, vindesine, vinorelbine, and vincamine within different domains of comprehensive information on these molecules such as their medical applications (contemporary/traditional), mechanism of antitumor action, and potential scale-up biotechnological studies on an in-vitro scale. The comprehensive information provided in the review will be a valuable resource to develop an effective, affordable, and cost effective cancer management program using these alkaloids.

**Supplementary Information:**

The online version contains supplementary material available at 10.1186/s12935-022-02624-9.

## Introduction

Cancer, a rapid formation of abnormal cells in an uncontrolled manner due to various modifications in gene expression, is one of the leading illness-related deaths worldwide [1–3]. In 2020, it accounted for nearly 10 million deaths globally, comprising a major portion of breast cancer > Lung cancer > Colon and rectum cancer > Prostate cancer > Skin cancer > and Stomach cancer related new cases (https://www.who.int/news-room/fact-sheets/detail/cancer). Today, chemically derived drugs treatment along with chemotherapy and radiotherapy constitute different components of cancer treatment, with chemo being adverse to healthy cells [4–6]. A comprehensive treatment of cancer is usually available in 90% of high-income countries, but it is only 15% in low-income countries, thus putting a tremendous economic cost on treatment (WHO, 2020). In 2010 alone, the total economic cost of cancer was estimated to be US$ 1.16 trillion [7]. Nevertheless, many cancers have a high chance of being cured if diagnosed early and treated appropriately [8, 9]. A 30–50% of cancers can be prevented by avoiding risk factors and implementing evidence-based preventive strategies (https://www.who.int/news-room/fact-sheets/detail/cancer).

For cancer treatment, plant secondary metabolites as chemo-preventive agents are well classified and recognized as bioactive compounds for primary and secondary prevention [10–12]. Consequently, many pieces of evidence prove that higher consumption of secondary metabolites can lower cancer development [13, 14]. These compounds have a regulatory effect on metabolic and signaling pathways, thus controlling the angiogenesis, and inhibition of microtubule assembly formation in cells and its apoptosis [15]. The secondary metabolites can be broadly classified into alkaloids, terpenes, flavonoids, lignans, steroids, curcumins, saponins, phenolics, and glucosides [16]. These secondary metabolites, either in individuals or in a group can be used for designing personalized cancer prevention programs as these plants based bioactive compounds present much required geno-protective effects such as DNA damage protection in healthy cells [17–19]**.**

In the class of phytochemicals, alkaloids have been promising anticancer agents. The alkaloids represent a highly diverse group of compounds, around 3000 distinct alkaloids have been characterized from plants, fungus, and animals together [20]. Some of the commonly known alkaloids include Morphine and Nicotine [21]. The alkaloids are low molecular weight organic nitrogenous compounds, often chemically classified into pyrrolidines, pyridines, tropanes, pyrrolizidines, isoquinolines, indoles, quinolines, and terpenoids and steroids. Generally, the alkaloids are colourless, crystalline, and non-volatile (https://www.britannica.com/science/atropine) and are reported as low in toxicity, with higher stability. It has been found that alkaloids impart a restraining effect on the topoisomerase enzyme, thus stalling DNA replication and cell death [22]. Therefore, alkaloids have been a base for drug development for various ailments such as anti-inflammatory, antibacterial, and antitumor [23]. The plant-based alkaloids have proven efficacy in oncogenesis suppression.

This review deals with different anticancer alkaloid compounds viz., (i) colchicine, (ii) vinblastine, (iii) vincristine, (iv) vindesine, (v) vinorelbine, and (vi) vincamine within different domains of existing information on these molecules such as their medical applications (contemporary/traditional), mechanism of antitumor action and potential scale-up biotechnological studies on in-vitro studies. The review shall be a valuable resource for the development of plant-based anticancer therapy, comprising different alkaloids.

## Review methodology

Electronic databases such as PubMed/MedLine, Science Direct, and TRIP database have been verified for preclinical pharmacological studies with included molecular mechanisms on the anticancer/cytotoxic/antiproliferative effects of isolated and identified natural alkaloids. Relevant high impact factors have been collected since December 2021. The following MeSH terms were used for the search: “Catharanthus”, “Phytotherapy”, Alkaloids/analysis”, “Alkaloids/isolation and purification”, “Alkaloids/therapeutic use’, “Alkaloids/pharmacology”, “Antineoplastic Agents/pharmacology”, “Apoptosis/drug effects”, “Autophagy/drug effects”, “Benzylisoquinolines/pharmacology”, “Cell Line, Tumor, Cytoprotection/drug effects”, “Humans, Signal Transduction/drug effects”, “Neoplasms/drug therapy”, “Neoplasms/ prevention and control”.

Studies published in English were included, which included plant-derived alkaloids with scientifically identified names, the type of cancer analyzed, the type of cell lines used in experimental pharmacological studies with molecular studies and molecular pathways of action highlighted. Abstracts, conference proceedings, studies that included homoeopathic preparations, and studies that showed pharmacological effects other than anti-cancer were excluded. The taxonomy of plants has been validated using the PlantList [24, 25].

## Traditional uses of natural alkaloids

### Colchicine

Colchicine is one of the oldest pharmaceutical medications. Dewar postulated the structure of colchicine in 1945 (Dewar, 1945), nearly a century after its first isolation in 1820, based on a degradation study by (Windaus, 1924). However, even though colchicine is one of the oldest medications currently in use, its metabolic effects and molecular actions are poorly understood. Colchicum corm has been used in the Unani System of Medicine for a long period for the treatment of arthralgia, gout, sciatica, etc. (Table 1).

**Table 1:** Traditional compound formulation, dosage, and use

Name of compound formulation and their forms	Type of Suranjan used	Administration	Indications	References
Roghan Gul Akh (Oil)	Suranjan Talakh (Colchicum luteum Baker)	Local application	Used as a poultice for gout and Rheumatic pains, Lumbago	[32]
Roghn Suranjan (Oil)	Suranjan Talkh (Colchicum luteum Baker)	Local application	Muscle pain, Sciatica, Gout	[32]
Roghan Waja al-Mufasil (Oil)	Suranjan Talkh (Colchicum luteum Baker)	Local application	Arthralgia	[32]

Pharmacological investigations have proven its effectiveness in different conditions reported by Unani Physicians, such as resolvent, deobstruent, sedative, aphrodisiac, phlegmagogue, but studies are required to prove it scientifically. It is nevertheless an important herbal treatment in the Unani System of Medicine, Unani Physicians utilize it sparingly because of its high toxicity. Ref. [26] used *Colchicum luteum* as the main ingredient of the formulation of Habb-i-Suranjan for curing pills, and investigated a case study along with regimental therapy (hot and moist fomentation applied locally) and found that Habb-i-Suranjan is quite safe and effective to cure osteoarthritis. Furthermore, colchicine, as a secondary therapeutic element, can be used to treat actinic keratosis by topically applying it in a cream base composition with an aminoglycoside antibiotic (e.g. gentamicin), without causing any toxicity or side effects. In the composition, Colchicine alkaloid (0.1–1.0% by weight) functions as a microtubule polymerization inhibitor. Colchicine has also claimed the cure different disorders and neurodegenerative illnesses based on the same mechanism of action [68]. There are several formulations such as laxative or purgative that are available in the market in which colchicine is used as a secondary drug [27], and formulations may include liquid, powder, suspension, spray, gel, tablet, capsule, etc.

The therapeutic use of colchicine has been better established in gout and familial Mediterranean fever (FMF). Apart from these, Colchicine’s potential therapeutic roles in rheumatoid arthritis (RA), and non-rheumatic illnesses such as pericarditis, atherosclerosis, and liver cirrhosis are still being investigated. The major alkaloid of colchicine, their action mechanism, mode of administration, dose as well as toxic effects was also discussed (Table 2).

**Table 2:** Properties of major alkaloid of *Colchicum autumnale*

Colchicine/traditional uses		Mode of action	Mode of administration	Doses	Toxicity/side effects	References
Alkaloid from ***Colchicum autumnale***	Powerful spindle poison anti-inflammatory used to treat a variety of conditions like gout, familial Mediterranean fever, pericarditis	Accumulates in leucocytes, interfering with leukocyte movement and degranulation, by binding tubulins, inhibits microtubule production	Oral drug delivery is unavailable the intravenous administration route is employed in dermatological problems, the topical pathway has been examined, and evidence of its effectiveness has been found	0.5–2 mg/daily per day, given once, twice, or three times a day	Gastrointestinal symptoms renal failure bone marrow suppression arrhythmias heart failure	[33–36]

### Vinblastine, vincristine, vindesine and vinorelbine

*Catharanthus roseus* is the most popularized medicinal plant that has been traditionally used as medicine since ancient times. The species contains more than 150 indole alkaloids out of 345 bioactive-phytochemicals, making it a well-known herbal medication with anti-cancer qualities (vinblastine, vincristine, vindesine) [28–30]. In the Ayurvedic system of medicine, various plant parts of *C. roseus* are used in folkloric herbal medicine for the treatment of cancer, gastrointestinal ailments, diabetes, liver, kidney, and cardiovascular illnesses all over the globe (Table 3). Traditionally, hot water extract of the dried leaves of the species has been used for curing diabetes in Jamaica, West Indies, however, hot water plant extract has been taken orally as complementary and alternative therapies for various syndromes such as heart problems, cancer in Peru [31]. Today, isolated indole alkaloids from *C. roseus* are available in the market under different trade names such as Velban, Oncovin, and Vinflunine [30]. These products are utilized for curing various diseases and syndromes like Hodgkin’s disease, Lymphosarcoma, Neuroblastoma, Carcinoma of the breast, etc. (Table 4).

**Table 3:** Worldwide traditional utilization of *Catharanthus roseus*

Plant parts	Preparation/extraction form	Mode of administration	Diseases	Country	References
Leaves	Dried and boiled with water	Oral intake	Menorrhagia, Diabetes	Australia	[37, 38]
Root bark	Dried and boiled with water	Oral intake	Febrifuge	Australia	[37, 38]
Whole plant	Dried and boiled with water	Oral intake	Diabetes	Brazil	[39]
Aerial portion	Dried and boiled with water	Oral intake	Menstrual regulators	China	[40, 41]
Whole plant	Dried and boiled with water	Oral intake	Diabetes	England	[30, 41]
Leaves	Dried leaf decocted	Oral intake	Diabetes	Europe	[40]
Whole plant	Boiled with water	Oral intake	Anti- galactagogue	France	[30, 41]
Whole plant	Boiled with water	Oral intake	Cancer, Hodgkin’s disease, menorrhagia	India	[30, 40]
Whole plant	Boiled with water	Oral intake	Diabetes	Pakistan	[42]
Whole plant	Dried plant decocted	Oral intake	Diabetes, liver disease	Taiwan	[43]
Leafy stem	Boiled with water	Oral intake	Diabetes	West Indies	[41, 44]
Whole plant	Powdered and mixed with cow’s milk	Oral intake	Diabetes	India	[31]
Root	Root air-dried, ground, and decocted	Oral intake	Urogenital infections	South Africa	[31]
Whole plant	Boiled with water	Oral intake	Diabetes, Hypertension, Dysentery, Cancer	Vietnam	[31]

**Table 4:** Trade name of Indole alkaloids in the market

Plant species	Indole alkaloid	Trade (Market name)	Use	References
*Catharanthus roseus*	Vinblastine sulphate	Velban	Hodgkin’s disease, Lymphosarcoma, Neuroblastoma, Carcinoma of breast	[41]
*Catharanthus roseus*	Vinblastine sulphate	Oncovin	Neuroblastoma, Wilkins’s tumor, Hodgkin’s disease	[41]
*Catharanthus roseus*	Vinblastine, Vincristine	Vinflunine	Anticancer	[45, 46]

The major alkaloids of *C. roseus* along with their mode of action, uses, toxicity, and side effects are mentioned in Table 5.

**Table 5:** The pharmacology of major alkaloids used for cancer treatment*,* their mode of action, oncological applications and side effects

Type of alkaloid		Plant part	Pharmacological mechanisms	Therapeutic indications	Side effects	Refs.
Vinblastine	Known as Vincaleukoblastine hygroscopic crystalline compound water/methanol miscible white/slightly yellowish colour	Leaf stem root	Clings to tubulin prevents microtubules from developing anti-mitotic	Breast cancer Lung cancer Head and neck cancer Hodgkin’s lymphoma Testicular cancer	↓Bone marrow ↑Gastrointestinal toxicity Potent vesicant Extravasation injury	[47]
Vincristine	Known as Leucocristine colourless fluid in most pharmaceutical formulations	Leaf stem root	Binds to tubulin dimer prevents microtubule structures from forming mitotic inhibitor anti-mitotic	Non-Hodgkin’s lymphoma lymphoblastic leukemia nephroblastoma	Peripheral neuropathy hyponatremia, constipation paralysis, spinal nerve demyelination, lung spasm	[48, 49]
Vindesine	*Catharanthus* alkaloid, brand names: Eldisine, Fildesin accessible as a dissolved powder, it has the appearance of a whitish fluid	Leaf stem root	Anti-mitotic	Melanoma Lung cancers Uterine malignancies	Spinal nerve demyelination, hyponatremia, constipation, hair loss, nerve demyelination, breathing problems, lung spasm	[49, 50]
Vinorelbine	It is the first 5ʹNOR derived semi-synthetic *Catharanthus* alkaloid; from *Catharanthus* alkaloids vindoline and catharanthine using Polonovski fragmentation method	Leaf stem root	Anti-mitotic	Breast cancer Non-small cell lung cancer	Inflammation of the veins, constipation, poor resistance to infection, bleeding, anaemia, nausea, diarrhoea, numbness or tingling in hands or feet	[51]
Vinflunine	Second-generation anticancer drug	Leaf stem root	↓ Transition from metaphase to anaphase, preventing cancer cells from entering mitosis ↑ apoptosis	Transitional cell carcinoma breast cancer	Hair loss Weariness Overall sensation of weakness	[52]

The pharmacological properties of the plant are also enumerated in Table 6.

**Table 6:** Biological activities of *Catharanthus roseus*

Activities	Plant parts	Plant extract	Bioactive compounds	Diseases type	Model/Test system	Results	Refs.
Anti-cancer	Stem leaves	Methanolic	Vinblastine Vincristine	Neoplasms, Chorio carcinoma Hodgkins disease	In-vitro Potato disc bioassay through *Agrobacterium tumefaciens* infection Positive control: camptothecin IC_50_ = 80.96, 82.68, 84.96% µg/mL	Inhibition concentrations: 10 ppm, 100 ppm, 1000 ppm	[53–55]
Anti-diabetic	Leaves flower	ethanolic	Vinculin	Streptozotocin-induced model diabetic rats	In vivo Swiss albino rats Dose = 50, 100, 200, 400 mg/kg, p.o Control: tolbutamide	↓ Blood sugar Hypoglycemic effect	[56, 57]
Anti-microbial	Leaves	ethanolic	Indole alkaloids Phenolic compounds	Bacterial cultures	In vitro *Pseudomonas aeruginosa, Staphylococcus aureus* *Bacillus subtilis* *Salmonella typhimurium* concentration of crude extract 1:20, 1:40, 1:60, 1:70, 1:80, 1:90 and 1:100	Antimicrobial agent	[58]
Anti-oxidant	Roots	Ethanolic extract	–	DPPH assay Superoxide radical scavenging	IC_50_ = 153.2 µg/mL	Antioxidant	[59]
Anti-helminthic		Ethanolic	–	Helmintic infections	In vivo *Pherithema posthuma* IC_50_ = 250 mg/mL Control: Piperazine	↓Growth	[60]
Anti-diarrheal	Leaves	Ethanolic	Flavonoids saponins	Anti-diarrheal	In vivo Wistar rats Dose = 200–500 mg/kg Standard: loperamide, atropine	↓Gastrointestinal transit	[61, 62]
Wound healing	Leaves	Ethanolic	Flavonoids triterpenoids	Incision wound model	In vivo Sprague Dawley rats Dose = 100 mg/bw daily	↑wound healing process	[63]
Hypo-glycemic activity	Leaves	Ethanolic	Vindoline Vindolicine Vindolin	PTP-1B inhibition ORAC DPPH pNPP	In vitro β-TC6 mice pancreatic cells C_2_C_12_ cells IC_50_ = 50 µg/mL	Hypoglycemic antioxidants	[64]
Hypolipidemic effect	Leaves	Juice	Vinpocetine	High diet	In vivo Guinea pigs Dose = 0.5–1 mL/bw	No significant change in lipid profile and body weight	[65]

## General characterization of natural alkaloids with anticancer properties

### Colchicine

**Fig. 1:**
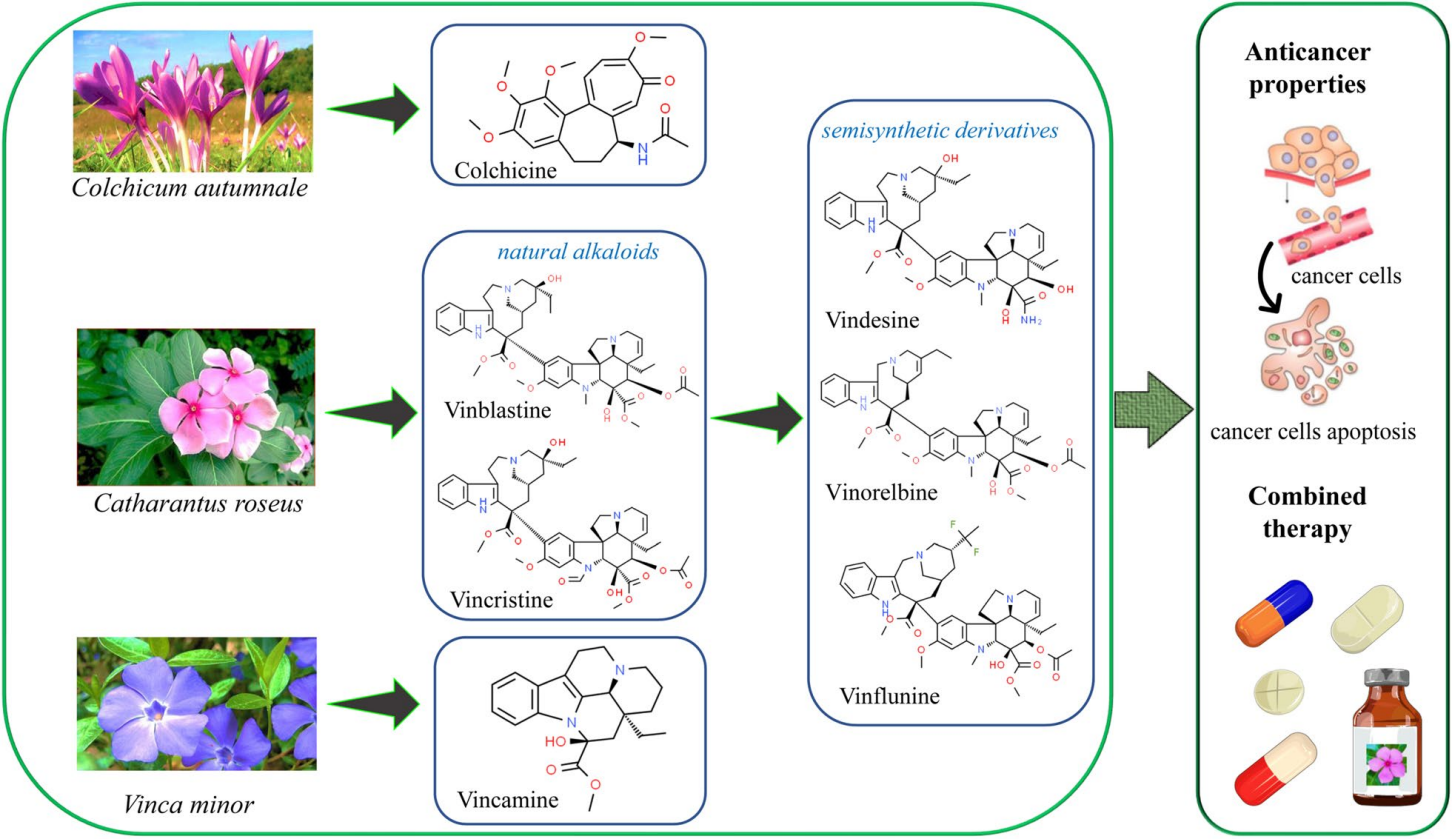
The chemical structures of the most representative natural alkaloids and their semisynthetic derivatives are used as anticancer agents

Colchicine is an alkaloid derived from the colchicum genus, derived from the autumn crocus (*Colchicum autumnale*) and Glory Lilly (*Gloriosa Superba*) plants, found primarily in Europe and North America (Additional file 1). Colchicine has been used in medications approved by the US Food and Drug Administration since 2009 [66]. It is a plant water-soluble alkaloid, odourless, with pale yellow needles or powder. The IUPAC name for Colchicine is *N*-[(7*S*)-1,2,3,10-tetramethoxy-9-oxo-6,7-dihydro-5*H*-benzo[a]heptalen-7-yl]acetamide with molecular formula of C_22_H_25_NO_6_ (https://pubchem.ncbi.nlm.nih.gov/compound/Colchicine) (Fig. 1). Colchicine is biosynthesized by phenylalanine and tyrosine which process 4-hydroxy-dihydro cinnamaldehyde (4-HDCA) and dopamine. A Pictet–Spengler reaction-based joining of 4-HDCA and dopamine results in a 1-phenethyl isoquinoline scaffold. This scaffold through a series of methylations and phenyl ring hydroxylations produces (S)-autumnaline. The (S)-autumnaline by phenol coupling and methylation results in *O*-methyl andro cymbine, which results in Colchicine by ring expansion and modification of nitrogen atom [67]. This water-soluble alkaloid has been most commonly used as a secondary treatment for gout, working as an anti-inflammatory agent. Derivatives of colchicine, like thio colchicine, deuterium enriched colchicine is also used for the treatment of pain or inflammation, bone-related disorders [68], Mediterranean fever (FMF), gout, scleroderma, secondary amyloidosis [69, 70], Behcet’s disease, pericarditis, coronary artery disease, and other inflammatory and fibrotic disease conditions [71]. Several reports demonstrated the efficiency of colchicine in chronic infections, autoimmune, allergic, cardiovascular syndrome, and neurodegenerative diseases [68, 72, 73]. The colchicine drug prevents DNA synthesis and tubulin polymerization, thus halting mitosis [74].

### Vinblastine, vincristine, vindesine and vinorelbine

#### Vinblastine

Vinblastine is a natural Vinca alkaloid that was initially identified from *Catharanthus roseus* (Fig. 1) (Additional file 1). When the extract was administered to rabbits to explore the plant’s putative anti-diabetic function, its potential as a chemotherapeutic agent was first revealed. In the experiment, rabbits died of a bacterial infection due to a lack of white blood cells, and vinblastine was therefore thought to be beneficial against malignancies of the white blood cells, such as lymphoma [75]. Since then alkaloids have become clinically useful for their antitumor properties. Due to putative hypo-glycemic properties, initially, it was isolated from extracts of *Catharanthus roseus.* It is structurally similar to two compounds of multi-ringed units: vindoline and catharanthine (https://pubchem.ncbi.nlm.nih.gov/compound/Vinblastine). It clings to tubulin and prevents microtubule formation, causing mitotic spindle assembly to be disrupted and tumour cells to be arrested in the M phase of the cell cycle. Bio molecules like amino acid, cyclic AMP, and glutathione metabolism; calmodulin-dependent Ca++-transport ATPase activity; cellular respiration; and nucleic acid and lipid biosynthesis may all be affected by this molecule. (NCI04). It’s used to treat Hodgkin’s and non-Hodgkin lymphomas, breast cancer, Kaposi sarcoma, renal cell carcinoma, and testicular cancer. Due to adverse reactions, it causes typical side effects such as myelosuppression, mucositis, fever, anemia, and alopecia.

#### Vincristine

Vincristine, also known as leurocristine, is a Vinca alkaloid with formula C_46_H_56_N_4_O_10_ obtained from *Catharanthus roseus* (Madagascar periwinkle) (Fig. 1) (additional file 1). It appears as a white crystalline solid with a melting point of 218 °C. The IUPAC name of the compound is methyl (1*R*,9*R*,10*S*,11*R*,12*R*,19*R*)-11-acetyloxy-12-ethyl-4-[(13*S*,15*S*,17*S*)-17-ethyl-17-hydroxy-13-methoxycarbonyl-1,11 diazatetracyclo[13.3.1.0^4,12^.0^5,10^]nonadeca-4(12),5,7,9-tetraen-13-yl]-8-formyl-10-hydroxy-5-methoxy-8,16 diazapentacyclo[10.6.1.0^1,9^.0^2,7^.0^16,19^]nonadeca-2,4,6,13-tetraene-10-carboxylate (https://pubchem.ncbi.nlm.nih.gov/compound/Vincristine). It is commonly used as a chemotherapy drug for the treatment of leukemia, lymphoma, myeloma, breast, head, and neck cancer. It played a vital role as a tubulin modulator, a microtubule-destabilizing agent, a plant metabolite, an antineoplastic agent, and a drug. It is a methyl ester, acetate ester, and tertiary alcohol, a member of formamides, an organic hetero-pentacyclic, and organic hetero-tetracyclic compound. It’s injected as an intravenous infusion and can be used in a variety of chemo programs. Its principal applications include non-lymphoma Hodgkin’s treatment with CHOP, Hodgkin’s lymphoma treatment with MOPP, COPP, BEACOPP, or the Stanford V chemotherapy treatment in acute lymphoblastic leukaemia (ALL), and nephroblastoma treatment. It’s likewise utilized with dexamethasone and l-asparaginase to promote recovery in ALL, as well as in combination with prednisone to manage juvenile leukaemia. Vincristine is utilized as an immunosuppressant in the treatment of thrombotic thrombocytopenic purpura (TTP) and chronic idiopathic thrombocytopenic purpura (CITP).

#### Vindesine

Vindesine is a Vinca alkaloid obtained from vinblastine that is being used to treat a variety of cancers, the most common of which being acute lymphocytic leukemia (Additional file 1). It works by preventing cells from entering metaphase mitosis by inhibiting tubulin mitotic function. The molecular weight of compound is 753.9 and molecular formula is C43H55N5O7 having IUPAC name, methyl (13*S*,15*S*,17*S*)-13-[(1*R*,9*R*,10*S*,11*R*,12*R*,19*R*)-10-carbamoyl-12-ethyl-10,11-dihydroxy-5-methoxy-8-methyl-8,16-diazapentacyclo[10.6.1.0^1,9^.0^2,7^.0^16,19^]nonadeca-2,4,6,13-tetraen-4-yl]-17-ethyl-17-hydroxy-1,11-diazatetracyclo[13.3.1.0^4,12^.0^5,10^]nonadeca-4(12),5,7,9-tetraene-13-carboxylate (https://pubchem.ncbi.nlm.nih.gov/compound/Vindesine) (Fig. 1). Vindesine is used to treat non-small cell lung cancer and juvenile chronic lymphocytic leukemia that is resistant to vincristine. Vindesine prevents cells from entering metaphase mitosis. In vitro experiments, it is three times more effective than vincristine and approximately ten times more effective than vinblastine at concentrations intended to block 10 to 15% of cells from entering mitosis. At tested doses that block 40 to 50% of cells in mitosis, vindesine and vincristine are nearly equal. In contrast to vinblastine, vindesine produces a small number of post-metaphase cells. Vindesine has shown promise in patients who relapsed after receiving vincristine as part of a multi-agent treatment regimen.

#### Vinorelbine

Vinorelbine suppresses cell proliferation by attaching to tubulin, and this distinguishes it from other alkaloids in its field of anticancer activity. Slowing of the growth rate of microtubules, an increase in the length of growth and a decrease in the shortening period are all evidence obtained in vitro on the dynamic instability of cancer cells [76].

### Vincamine

Vincamine is a monoterpenoid indole alkaloid isolated from the foliage of *Vinca minor* (lesser periwinkle). It accounts for around 25–65% of the indole alkaloids in Vinca minor (Additional file 1). The molecular formula of compound is C_21_H_26_N_2_O_3_ and IUPAC name, methyl (15*S*,17*S*,19*S*)-15-ethyl-17-hydroxy-1,11 diazapentacyclo [9.6.2.0^2,7^.0^8,18^.0^15,19^] nonadeca-2,4,6,8(18)-tetraene-17 carboxylate (https://pubchem.ncbi.nlm.nih.gov/compound/VINCAMINE) (Fig. 1). The alkaloid is often used to prevent and cure cerebrovascular diseases and inabilities. It has pharmacological properties in both the central nervous system and the cardiovascular system, but its principal effect is on the brain vessels. It is often used for circulatory disorders, cerebral circulatory illness, assistance for brain metabolism and improved oxygen supply, strengthen prophylaxis, concentration impairment and betterment of memory and cognitive capacity, psychological productivity, prevention of aging of brain cells, improvement of immune function, diarrhoea, throat ailments, vaginal flux, tonsillitis, sore throat, intestinal inflammation, toothache, dropsy, diuretic, and blood-purifying and blood-purifying and wound healing.

## Anticancer/cytotoxic potential of alkaloids: pharmacological mechanisms of actions

### Colchicine

#### Mechanism of antitumor action

**Fig. 2:**
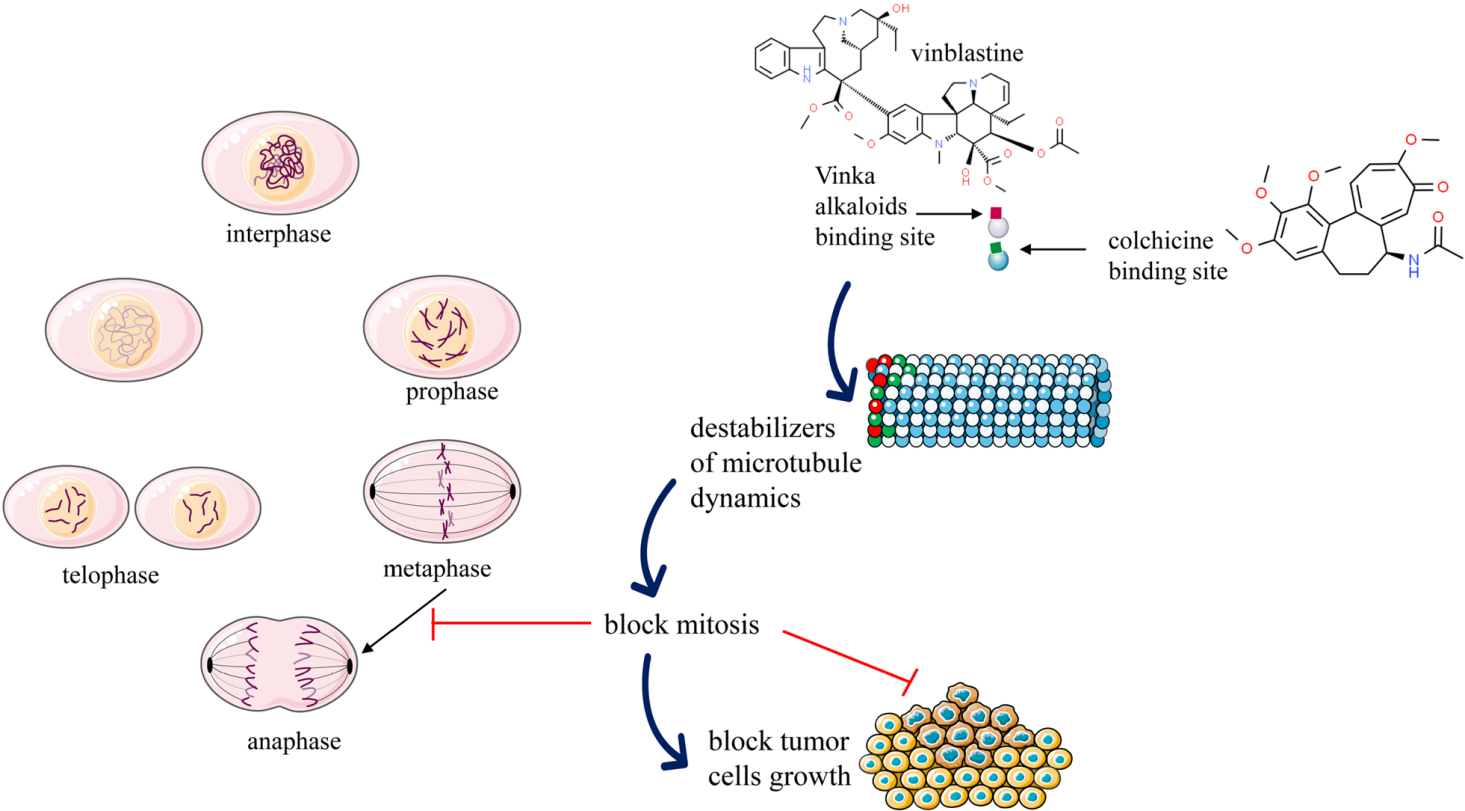
Summarized scheme with anticancer mechanisms of Vinca alkaloids and colchicine. These compounds stop the mitotic cell cycle by attaching to the surface of two tubulin heterodimers next to the exchangeable GTP-binding site, causing microtubule depolymerization. Destabilization and interruption in tubulin dynamics result in programmed cell death or apoptosis

Colchicine extracted from *Colchicum autumnale* binds to microtubular ends and inhibits microtubule polymerization. Colchicine is a well-known anti-mitotic medication that prevents mitotic cells from entering metaphase (Fig. 2). One of the most common therapeutic actions of colchicine is its capacity to bind to tubulins [71]. Microtubules are composed of αβ-tubulin heterodimers, key components of the cytoskeleton. Colchicine interacts with soluble tubulin to create the tubulin-colchicine (TC) complex, which combines microtubular ends and disrupts tubulin lattice dynamics [77–79]. Colchicine inhibits assembly by preventing the curved tubulin from adopting a straight structure. Interaction of colchicine and cell lipid membrane results in alterations in properties of compound and membrane [80]. High and low concentrations of colchicine promote and arrest microtubular polymerization and growth, respectively [71]. Colchicine also inhibits cancer cell movement and metastatic potential, cell blebbing via the Rho/Rho effector kinase/myosin light chain kinase pathway, a restricted input of ATP into mitochondria, and the release of caspase protease and cytochrome-*c*, resulting in cell apoptosis [81, 82].

Tubulin disruption and anti-mitotic effect of Colchicine: Colchicine can bind to tubulins, preventing microtubule formation and polymerization [68]. Microtubules (important components of the cytoskeleton), which are made up of -tubulin heterodimers are engaged in a variety of cellular processes such as cell migration, cell division, ion channel control, cell shape, and so on [71]. Colchicine is involved in the inhibition of cancer cell migration and metastatic potential [82], and inhibition of angiogenesis [81]. Colchicine possesses anti-inflammatory properties, which are mostly due to the disruption of microtubules and leucocyte downstream cellular activities.

#### Scientific studies related to anticancer properties

The antitumor effect of colchicine was investigated on hypo-pharyngeal cancer. In a dose-dependent way, colchicine suppressed the development and proliferation of human hypo pharyngeal cells. Results have shown that colchicine suppressed cell invasion, cell migration, adhesion via* MMP9*, *uPA*, and *FAK/SRC* reduced expression. Suppression of cell movement and invasion of hypo-pharyngeal cells occurs by reduction of ECM degradation via *MMP9* and *uPA* system down-regulation. Colchicine induced cell apoptosis and inhibited cell metastasis in hypo pharyngeal cell lines. Cell apoptosis is induced by colchicine through caspase-3 activation [83].

Colchicine in a dose-dependent manner has been reported for its antitumor effect on gastric carcinoma cell lines i.e., AGS and NCI-N87. Colchicine stimulated cell apoptosis in NCI-N87. Apoptosis development in the cells includes a series of changes which contains anti-apoptotic Bcl-2 protein, blockage of cytochrome *c* release, pro-apoptotic Bax protein releases cytochrome *c* which in turn activates caspase-3 and 9 and are key triggers of apoptosis executors. In conclusion, colchicine bars the expression of the Bcl-12 protein however it increases Bax, cytochrome *c*, and caspase-3 concentrations, resulting in cell apoptosis. Colchicine promoted caspase-3-mediated apoptosis through the suppression of the PI3K/Akt/mTOR signaling pathway in NCI-N87 cells [84–86]. Ref. [87] reported the anti-proliferative effect of colchicine on AGS and NCI-N87 cell lines at an optimal dose concentration of 6 ng/mL, up-regulating 18 genes and down-regulating 12 genes. Among the up-regulated genes, *DUSP1* was the sole up-regulated gene that contributed to colchicine’s anti-proliferative action on gastric cancer cells. Upregulation of DUSP1 resulted in the repression of the extracellular signal-regulated kinase, S-phase kinase-associated protein 2/CDC28 protein kinase 1B ubiquitin ligase complex, as well as the reduction of cell proliferation and initiation of cell death. When compared to control mice, colchicine-treated mice showed decreased tumor volume ratios and slow overall tumour development rates after 2 weeks of treatment. Colchicine derivatives reduced P-gp-induction activity by changing its conformation and have a potent cytotoxic effect against two colon cancer cell lines i.e., HCT-116 and Colo-205. Colchicine derivatives have also inhibited microtubular assembly and induced the cell expression of pro-apoptotic protein p21 [88].

Investigation of colchicine against human breast adreno-carcinoma MCF-7 cells demonstrated that colchicine induced the cell cycle arrest at the G2/M phase of mitosis along with down-regulation of MMP-2 mRNA expression in MCF-7 cells [89].

### Vinblastine, vincristine, vindesine and vinorelbine

The stem and leaves of *C. roses* are rich in Vinca alkaloids i.e., vincristine, vinblastine, and semi-synthetic derivatives viz. vindesine, vinorelbine, and vinflunine [90, 91]. Vinflunine and vinorelbine have been produced to increase the therapeutic action and synthesized using the precursor alkaloids catharanthine and vindoline. Vinblastine has been used specifically in the treatment of neoplasms, Hodgkin’s disease, and carcinomas. Vincristine has been specifically employed in leukemia in children [54]. Vinflunine and vinorelbine have been investigated for transplantable murine and human tumors, second-line transitional cell carcinoma of the urothelium [92, 93]. These compounds show antitumor activity by binding to tubulin and are generally known as ‘mitotic poison’. The cell cycle is arrested at the metaphase of microtubules in mitosis [42, 92].

#### Potential use as an anticancer agent

*Catharanthus roseus* has been found to contain a wide range of alkaloids (viz., vinblastine, vincristine, vindoline, vindolidine, vindolicine, vindolinine, and vindogentianine) which possess anticancer properties [64, 94]. They have the capabilities to inhibit cell proliferation through changing the microtubular dynamics, which induces apoptosis [95]. Hodgkin’s disease, choriocarcinoma, neuroblastoma, lymphosarcoma, and carcinomas of the breast and lungs have all been treated with vinblastine sulphate. While the vincristine sulfate, an oxidized form of vinblastine [95], blocks mitosis in the metaphase and helps treat acute juvenile leukemia, lymphocytic leukemia, Hodgkin’s disease, reticulum cell sarcoma, neuroblastoma, and Wilkins’s tumor [41].

Vinblastine’s anticancer activity is considered to be caused to its interaction with tubulin, which inhibits mitosis at metaphase. Vinblastine attaches to the mitotic spindle’s microtubular proteins, causing microtubule crystallization and mitotic standstill or inducing apoptosis [94, 95]. Vinblastine and its derivatives as anticancer medicines have about the same affinity locations to gain efficacy towards cancer cells through a double-sided sticking process that interacts with α and β-tubulin [86]. Additionally, Vinblastin’s predecessor in terms of chemistry, Vindoline is a cancer-fighting compound too made from tabersonine [96].

Likewise, Vincristine is also a Vinca alkaloid that occurs naturally and is an organic molecule [97]. This alkaloid (vincristine drug) acts as an anti-microtubule agent that blocks mitosis by blocking cells in the metaphase [95, 98]. These medications work by blocking tubulin polymerization leading to the halted synthesis of microtubules, as well as triggering depolymerization of tubules that have already formed. Interfering with nucleic acid and protein synthesis by preventing glutamic acid utilization is another method of vincristine activity [96]. Acute lymphocytic leukemia, lymphoid blast crisis of chronic myeloid leukemia, and Hodgkin and Non-Hodgkin lymphoma are among the applications for vincristine approved by the US Food and Drug Administration (FDA). Central nervous system malignancies, Ewing sarcoma, prenatal trophoblastic tumours, multiple myeloma, ovarian cancer, and small cell lung cancer are among the off-label applications of vincristine [97, 98].

#### Mechanism of antitumor action of substances

Vinca alkaloids present in *C. roseus* alter the microtubular dynamics leading to cell growth regression and apoptosis. Microtubules are cytoskeleton mitotic spindle components that help the cell separate its chromosomes during mitosis and meiosis (Fig. 2). Microtubules are essential for cell structure, transport, and a variety of other cellular processes [99]. Microtubules are heterodimers made up of α-and β-tubulin that polymerize and depolymerize dynamically at their ends. Microtubules assembly and disassembly are known as ‘treadmilling’ and ‘dynamic instability, respectively, and are controlled by guanosine triphosphate and tubulin binding [91].

These complexes Vinca alkaloids and tubulin heterodimers are stabilized by electrostatic and Van-der Waals interaction at the common binding site. Basically, two groups of destabilizing compounds have been differentiated clearly, first involves preventive depolymerization called microtubules stabilizing agent; the second is microtubulin destabilizing ones inhibiting microtubulin assembly formation [100]. The presence of two fluorine in vinflunine improved the electrostatic binding compared to vinorelbine. The catharanthine domain is responsible for the cytotoxic effect, whereas tubulin heterodimer binding is through the vindoline domain [101]. Vinca alkaloids and their derivatives have varying affinity due to distinct equilibrium constants: vincristine > vinblastine > vinorelbine > vinflunine [91]. Another mechanism of action of Vinca alkaloids involves their interplay with microtubule-associated proteins, interaction with calmodulin, and thus, inhibiting amino acid metabolism [102]. Different Vinca alkaloids have different modes of action, which might harm their activity. Such as, despite having a lower binding affinity for tubulin than vinblastine or vincristine, the variation in the effectiveness of vinflunine relative to vinblastine is best explained by its interaction with calmodulin [95]. Recently, cell growth inhibitors also have been reported from *C. roseus* however, their mode of action is still not very clear.

A new monoterpenoid indole alkaloid from *C. roseus*, Catharoseumine has a distinctive peroxy bridge and showed a cytotoxic effect against human promyelocytic leukemia HL-60 cell lines [31, 99]. Another new bis-indole compound from cathachunine showed a selective inhibitory antitumor effect on human leukemia cells [31].

##### Natural vinca alkaloids: vinblastine and vincristine

Vinblastine and vincristine particularly arrest the metaphase of the cell cycle due to cell interaction and disruption of microtubule function and tubulin comprising of mitotic spindle apparatus [90] (Fig. 2). The inhibition of cell proliferation by vinblastine which binds to the microtubule ultimately causes mitotic block and apoptosis [103]. Each microtubule comprises 16–17 binding sites, which are localized at the microtubule’s ends. Vinblastine and vincristine bind to these sites interrupting the microtubular congregation, declining the growth rate along with microtubule shortening at the assembly end, producing kinetic cap, and suppressing its function [104]. The microtubular dynamic disturbance by vinblastine particularly at the ends of the mitotic spindle and metaphasic arrest generally occurs at low drug concentrations [105]. Vinca alkaloids are known to show effects on both malignant and non-malignant cells in which microtubules have a role in the non-mitotic process in the non-mitotic cell cycle [90, 106]. Vinblastine disrupts microtubules, inhibits protein and nucleic acid synthesis, raises oxidized glutathione and cyclic adenosine monophosphate (cAMP) levels, inhibits calcium-calmodulin-regulated cAMP phosphodiesterase, and changes the membrane’s lipid composition [107, 108]. Antineoplastic activity of Vinca alkaloids is mainly attributed to its ability to inhibit micro spindle formation, dissolution of the mitotic spindle, and diving cells’ metaphase arrest. In the cell, morphological changes and its death were reported at interphase and G and S-phase in normal, leukemic lymphocytes and cultured leukemic cells [107]. The antitumor effect of vinblastine and vincristine is caused by their binding to intracellular tubulin, inhibiting DNA repair and RNA synthesis by inhibiting DNA-dependent RNA polymerase enzyme. The cellular processes like glycolysis, respiration, nucleic acid, and protein synthesis [108, 109]. Vinblastine and vincristine being hydrophobic when uncharged, can easily partition into lipid bilayers thereby, modifying structure along with the function of the membrane [110].

Vinblastine is used in lymphomas, bladder, and breast cancer treatment whereas, in acute lymphoblastic leukemia during early clinical trials, their complete remission is released. The neurotoxicity limits the dose of vincristine and vinblastine is generally used as a single dose agent hence, given in higher doses [108]. However, in combinational therapy, vincristine is used as many chemotherapies are myelosuppressive similar to vinblastine.

##### Semisynthetic derivatives: vindesine and vinorelbine

Vindesine had been the first analogue of vinblastine for clinical use. Vindesine bears an amide functional group compared to ester in vinblastine and lacks an acetyl group on the vindoline ring. Vindesine is administered in combinational therapy for the management of leukemia, non-small cell lung cancer, and lymphoma [111]. The mechanism of action of vindesine like other Vinca alkaloids involves its binding to microtubular protein tubulin, metaphasic cell cycle followed by prevention of polymerization of tubulin for the formation of micro spindle and depolymerization of formed microtubules. The lower hepatotoxicity of vindesine has made it preferable over vinblastine in combination drug therapy [111, 112].

Vinorelbine is a semi-synthetic Vinca alkaloid derivative, a mitotic spindle poison impairing chromosomal segregation, which blocks cells at the G2/M phase during mitosis. By opening the catharanthine ring, vinorelbine establishes a reversible covalent bond with tubulin (8-membered) [113]. Vinorelbine promotes the apoptosis of cells and disorganizes the mitotic spindle resulting in the stimulation of the tumour suppressor gene p53, as well as the activation/deactivation of various signal pathways such as WAF1/CIP1, Ras/Raf, PKC/PKA, and the inactivation of the apoptosis suppressor Bcl2 [103, 114]. This inactivation induces a decrement in the creation of hetero-dimer between Bcl2 and the pro-apoptotic gene BAX which stimulates cell apoptosis [103]. Vinorelbine causes mitotic slippage failing cells to be in the metaphasic phase for an extended time and their DNA replication without cytokinesis and cell death known as ‘mitotic apoptosis’, ‘mitotic catastrophe’ or ‘abortive mitosis’ [115].

#### Scientific studies confirmed the anticancer properties

##### Vinblastine

Vinblastine is an alkaloid that is being used to treat neoplasia, resistant pregnancy choriocarcinoma, metastatic testicular tumours, breast cancer, Kaposi carcinoma, and Letterer–Siwe illness [29]. In the P388 murine leukemia model, HeLa, and MCF-7 (breast cancer) cell lines, vinblastine derivatives exhibited potential anti-tumor activity [116]. In non-small cell lung cancer, melanoma, breast cancer, colon cancer, and P388 and L1210 leukemia, nitro derivatives of amino vinblastine have anticancer action. Shao et al. [117] observed human non-small lung cancer and human cervix epithelial adenocarcinoma cell lines treated with carbamate derivatives of vinblastine displayed anticancer activity. Similarly, the growth of HeLa cells was inhibited by amide substituted anhydrous vinblastine derivatives [116]. Vinblastine promotes acute, cell cycle phase-independent acute apoptosis in certain lymphomas including ML-1, and chronic lymphocytic leukemia. A conjugate consisting of adenocarcinoma-reactive MAb KSI/4S2, on coupling its lysine amino group with a 4-hydroxy group of 4-desacetyl vinblastine through a succinate bridge, which contains 4–6 desacetyl vinblastine molecules per molecule of KSI/4S2, demonstrated a significant antitumor effect in vivo against human lung and colorectal adenocarcinoma xenografts in nude mice [118].

##### Vincristine

Crude methanolic extracts of vincristine in combination therapy with conferone from *Ferula schtschurowskiana* increased its cytotoxicity in the cancer cells. Conferone competitively binds to P-gp transporters, decreases the vincristine resistance, thus, and enhances vincristine cytotoxicity [119, 120]. In-vitro and in vivo clinical studies of vincristine sulphate demonstrated an increase in cell death when administered intraperitoneally. Vincristine sulphate binds to malignant cells, cell proliferation inhibition by altering tubulin dynamics and affects cellular processes DNA, RNA, lipid biosynthesis, glutathione metabolism, and calmodulin-dependent ATPase activity [121]. In a study, on vincristine sensitive and resistant HeLa cervical carcinoma and MCF-7 breast cancer cells, the anticancer impact of microtubule destabilizing drugs’ vincristine in conjunction with siramesine was examined. The death of vincristine-treated HeLa within 48 h went with G2-M cell cycle arrest, lysosomal membrane permeabilization, cytochrome *c* release, Bax and caspase initiation, and apoptosis-like chromatin condensation [122]. However, MCF-7 cells opted for mitotic arrest, reattached to bottom cells with metabolic activity with the intact plasma membrane, and were viable for many days. Vincristine promotes mitotic cell cycle seizure in SH-SY5Y human neuroblastoma cells by regulation of cyclin B and D and apoptosis via stimulation of caspase-3 and -9 [123]. Vincristine to minimize drug resistance given in combination therapy with suberoyl anili-dehydroxamic acid (SAHA) is effective on T cell lymphoblastic leukemic cells. Vincristine in combination with SAHA causes a change in microtubule dynamics through HDAC6 inhibition resulting in M phase arrest, an upsurge in the number of cells at the sub-G_1_ phase along with initiation of the apoptosis pathway [124].

##### Vindesine

Vindesine is a second-generation semi-synthetic Vinca alkaloid that possesses broad-spectrum antitumor activity in-vitro with lesser neurotoxicity. In mice, vindesine demonstrates the effect against Ridgeway osteogenic sarcoma, Gardner lympho sarcoma, P154 leukemia, and P388 leukemia, as well as murine B16 melanoma and S180 ascites tumour in preclinical investigations [112]. Vindesine with methotrexate when administered peritoneally with L1210 leukemia cells reported a 200% increase in the life span of mice. In a randomized phase III study, vindesine in conjugation with Epirubicin did not increase the efficacy of the drug [125]. In another study, patients with advanced breast cancer were randomized given vindesine (3 mg/m^2^) with Adriamycin gave an overall response of 63% with survival of 43 weeks [126]. Ref. [127] reported single-agent vindesine (3 mg/m^2^) to 63 patients with inoperable non-small cell lung cancer resulting in a 14% response rate compared to vincristine with 0%.

##### Vinorelbine

Vinorelbine is proven to be efficacious in glial tumours with low proliferation attributes [115]. In pediatric diffuse pontine glioma, a combination of vinorelbine and nimotuzumab (anti-EGFR monoclonal antibody) has proven to be a successful compound [115]. In another study, [128] suggested treating juvenile patients with progressing optic pathway glioma with a single dose of vinorelbine. The overall treatment showed low toxicity along with good life quality. Vinorelbine may synergize with immune therapy in treating severe malignancies as it unaccompanied triggers inflammatory immunity and pontine gliomas may, in turn, react to immunotherapy treatments. Individuals with malignant glioma and pontine glioma who were treated locally with IL-2-stimulated Natural Killer cells had their Karnofsky performance score increased from 20 to 60.

A phase II trial published in 2012 revealed that PEGylated IFN-2b therapy slowed the progression of diffuse intrinsic pontine glioma in children. Tuna et al. [129] found that vinorelbine produced considerable cell death in C6 glioma cells and inhibited DNA synthesis in glioma spheroids. In MCF-7 human breast cancer cells, vinorelbine activates ERK2 [115]. Shi et al. [130] found that phospho-ERK1/ERK2 expression is linked to better relapse-free survival in lung cancer patients treated with vinorelbine in both univariate and multivariate statistical tests. In-vivo growth of human cancer xenografts was suppressed by a combination of vinorelbine and ERK inhibitors. In the first line of treatment for metastatic breast cancer, a combination of vinorelbine and docetaxel has shown a significant tumour response with acceptable toxicity [131]. Vinorelbine has been effective in the treatment of breast cancer owing to its strong affinity for mitotic tubulin, and low degree of neurotoxicity, hence, making it a promising option for phase II clinical trials in patients with advanced breast cancer who have failed first- or second-line chemotherapy.

### Vincamine

#### Mechanism of antitumor action of substances

The mechanism of action involves tubulin polymerization inhibition and microtubule dynamic behavior interference in targeted cells causing cell arrest and apoptosis [132]. Apoptosis in Vinca alkaloids involves a pathway independent cell arrest. Additionally, tubulin binding agents involve calmodulin and signal pathways perturbation along with phosphorylation of Bcl-2, Raf-1 kinase, Bcl-xL, and p53 [132, 133]. Intracellular mitochondrial apoptosis is mediated by caspase-3 causing the breakdown of many cellular proteins. Vincamine has a strong affinity for caspase-3 hence, promoting caspase-3 apoptosis [134]. Apoptosis action of Vincamine causes a reduction in mitochondrial membrane potential followed by disruption of mitochondrial transmembrane resulting in cytochrome G release in the cytosol and subsequently caspase-3 activation causing a decrease in cell viability [135]. Mechanism of the antitumor potential of Vincamine involves activation of various signal pathways i.e., decrease in iron intracellular concentration in dividing cells, triggering cell death by disrupting mitochondrial membrane, and direct activation of caspase-3 [134].

#### Scientific studies confirmed the anticancer properties

Vincamine treatment is used against lung cancer due to its unique functions in cancer cell apoptosis. Vincamine is a powerful antioxidant that neutralizes hydroxyl free radicals, promotes the Nrf2/HO-1 signaling pathway, and suppresses NF-B-mediated iNOS production. Vincamine suppresses cancer cell growth by lowering iron levels in cancer cells. The iron-responsive element/iron regulatory protein (IRE/IRP) regulatory system balances iron levels in cells, and vincamine targets this system to lower iron levels in lung cancer cells, trigger caspase-3, and induce cell death by interrupting the mitochondrial membrane [134].

## Other medical applications of alkaloids

### Colchicine

#### Colchicine and immune system

Nuki’s 2008 discoveries [136], in understanding the pathophysiology of crystal-induced inflammation have revealed novel insights into colchicine’s molecular mechanisms in crystal-associated arthropathies (Crystal-associated arthritis is often seen in association with signs of inflammation and degenerative joints in middle and old age humans). Inhibition of neutrophil chemotaxis, adhesion, mobilization, and superoxide production, as well as inhibition of NALP3 (NACHT-LRRPYD-containing protein 3) inflammasomes and interleukin 1 processing and release, are the key actions for Colchicine based arthropathies [71, 136].

#### Colchicine and muscular functions

Colchicine is used to treat muscle diseases that functionally decrease microtubule-dependent NADPH oxidase-2 (NOX2) signaling, thereby improving muscular function. According to a report, a mixture of 5–20 g/kg colchicine and other compounds that block sarcolemmal calcium (Ca^2+^) channel activation and renin–angiotensin signaling can be given before, during, or after the onset of muscle damage or muscle soreness for the treatment of muscular disorders by reducing overall injury, exercise fatigue, and hence improving muscular strength [68].

### Vinblastine, vincristine, vindesine and vinorelbine

#### Antidiabetic

*Catharanthus roseus* has been traditionally used as a treatment for diabetes in several regions of the world. Several reports indicated that juice of leaves and whole plant of the species has capabilities to reduce glucose levels in the body and [94] reported that four indole alkaloids (vindoline, vindolidine, vindolicine, and vindolinine) isolated from plants showed antidiabetic properties via the assays of 2-NBDG glucose uptake and inhibition of PTP-1B, an enzyme that regulates negatively the insulin signalling pathway.

#### Neuroprotective

Few reports indicated that aqueous extract (plant parts) of *C. roseus* has been shown to inhibit AchE in an in-vitro microassay [137, 138]. Additionally, serpentine, an alkaloid present in *C. roseus* showed strong activity against AchE with a low IC50 value (0.775 µM) [138]. These reports suggested that plant is a potential source of active compounds for the pharmacological management of neurodegenerative conditions including Alzheimer’s disease.

## Design and development of anticancer agents based on natural alkaloids: an overview

The anticancer properties of natural alkaloids have been used traditionally and in contemporary medicine to advance cost-effective overall cancer management. For cancer treatment, researching a natural substance represents an attractive strategy [139]. As plant-based drug development is advancing due to its proven efficacy and cost-effectiveness, plant alkaloids are the most promising. For the discovery of new natural product-based chemopreventive agents, a judicious selection and a holistic approach for the selection of compound (individual or group), plant source, and plant part are pivotal [18, 140, 141]. In the scenario of tremendous anthropogenic pressure on natural plant resources and many plant resources coming into the category of “Rare” or “Endangered”, the task of getting the natural compounds becomes a tough job though [142]. Nevertheless, a sustainable approach regarding anticancer compounds can be, the harvest of novel alkaloid compounds/Biopharmaceuticals from different in-vitro techniques at an industrial scale.

Several drugs have been developed for cancer in recent years, these drugs come with the side-effects along with the development of drug resistance in patients with time [8, 9, 143, 144]. Therefore, it is difficult to synthesize a chemical-based drug that can distinguish between healthy and tumor cells effectively. Looking at the untoward effects of synthetic drugs, an upsurge, therefore, has been noted in the usage of plant-based drugs in developed and developing countries, mainly due to proven efficacy, safety, lesser side effects, accessibility, and acceptability [145, 146].

Colchicine is a typical anti-mitotic medication that binds to soluble tubulin to create tubulin-colchicine complexes in a weakly reversible manner, which subsequently adheres to the terminals of microtubules to prevent the microtubule polymer from elongating. Colchicine inhibits microtubule development at low concentrations but stimulates microtubule depolymerization at higher concentrations. At high doses, it causes significant damage to normal tissues, limiting its utility in cancer therapy [76]. Colchicine is an anti-inflammatory substance extracted from the leaves, flowers and seeds of saffron and has long been included in the prescription drug for cancer patients, but the same problem has always arisen: the drug attacks both cancer and healthy cells, destroying them [147]. In a recent study, the stabilization of colchicine was done by attaching a chain of amino acids that make it inert, and the colchicine molecule moves freely through the body in this state, without affecting the healthy cells it encounters. Once in contact with the tumor, the amino acid chain is removed by an enzyme present on the surface of the cancer, called MMP-1. Thus, Colchicine is activated and destroys the surrounding cancer cells. MMP-1 (matrix metalloproteinases) is an enzyme that plays a vital role in destroying the extracellular matrix. Tumours use it to grow and invade healthy tissue [148]. The enzyme increases blood flow around it, building new blood vessels and giving the tumor access to nutrients and oxygen. By destroying it, the resources needed for tumor growth and metastasis are cut off. The results of the study showed that the use of the enzyme MMP-1 as an activating agent helps the drug treat secondary tumors caused by cancer after it has spread in the body [148].

Vinblastine sulfate, USP is the salt of an alkaloid obtained from Vinca rosea Linn, the flowers of a common medicinal plant (known as *Catharanthus roseus* G Don). Before the design and development of this drug, the generic name was vincaleucoblastin, abbreviated VLB. It is a statokinetic oncolytic agent. In vitro treatment with this preparation stops the growth of cells at the metaphase level. Chemical and physical evidence indicates the empirical formula of vinblastine as C46H58N4O9H2SO4 and that it is a dimeric alkaloid containing both indole and dihydroindole in equal parts [149].

Vincristine is available as a lyophilisate for the preparation of a solution for intravenous administration [150]. However, the reduced bioavailability and dose-dependent neurotoxicity limit the therapeutic utility of this agent. As a result, incorporation into target transport pharmaceutical forms such as liposomes has been an important prospect. Injections with liposomes with vincristine sulfate, 0.16 mg/mL (Marqibo^®^) is a new formulation in which Vincristine is encapsulated in sphingomyelin and cholesterol nanoparticles, to increase the release and intensification of the therapeutic dose of Vincristine [150, 151].

Vindesine is marketed as vindesine sulfate and approved by the FDA in 1994. The mode of action of vindesine sulfate is not completely known [152]. Like other Vinca alkaloids, vinblastine sulfate and vincristine sulfate, vindesine sulfate block cells in metaphase during mitosis [90]. In vitro investigation has also shown that vindesine sulfate prevents malignant cells from invading normal tissue However, comparative studies with these three alkaloids have shown significant differences between their molecular effects. Vindesine sulfate is 3 times more potent than vincristine and almost 10 times more potent than vinblastine in blocking the effect of mitosis in tissue culture studies designed to block 10% to 15% of cells in mitosis [108]. At dose concentrations that block 40–50% of the cells in mitosis vindesine sulfate and vincristine are approximate equal potency and both have 3 times the potency of vinblastine. Also, qualitative differences were observed between the three alkaloids [108]. At low doses, vinblastine produces a predominance of cells in the post-metaphase, in which the center and chromosomes appear very obvious. Cells exposed to vincristine show spherical metaphase with compact chromosomes on a contracted spindle. Unlike vinblastine, vindesine sulfate produces few post-metaphase cells [153]. In cells exposed to vindesine sulfate, the spindles were inflated with scattered chromosomes, in stark contrast to the tightly-packed chromosomes observed with vincristine. Vindesine sulphate showed oncolytic activity in patients with relapses during polychemotherapy treatment that included vincristine. In laboratory animals and humans, the biliary system is the major route of excretion of vindesine sulfate [76].

Vinorelbine is another Vinca alkaloid that’s semi-synthetic and sold by the name Navelbine. It is a chemotherapeutic drug for treating non-small cell lung cancer that has spread metastatically (NSCLC) (https://pubchem.ncbi.nlm.nih.gov/compound/Vinorelbine). It is administered by intravenous injection or by mouth during the first-line therapy of advanced or metastatic NSCLC in combination with other drugs (e.g., cisplatin). It has been licensed for therapeutic usage in the United States in 1994 [154].

## Therapeutic limitations

Therapeutic limitations of alkaloids derive from their side effects and pharmacokinetic properties [155].

### Side effects of alkaloids cytostatic drugs [96, 152, 156]

i. Myelosuppression and other hematological effects. Leukopenia occurs in the 1st days after administration. Thrombocytopenia is mild and transient [157].
ii. Nausea and vomiting [158].
iii. Extravasation can lead to severe inflammation, pain and tissue damage [159].
iv. Alopecia, which is typical incomplete [160].
v. Neurotoxicity: Vinca alkaloids may cause central and peripheral neurotoxicity, including vegetative neurotoxicity. The risk of neurotoxicity may be increased by high doses or prolonged therapy. Neurotoxicity can occur days or weeks after starting treatment, with recovery usually weeks or months after stopping treatment [161]. Neurological effects are usually less common and severe than with vincristine. Mild paresthesias are the most commonly reported neurological toxicity and are usually reversible upon discontinuation of vinblastine. Other neurological toxicities may include numbness, neuritis, muscle cramps, loss of deep tendon reflexes, headache, malaise, weakness, dizziness, convulsions, depression, psychosis, severe pain in the face and jaw, severe immediate or delayed tumor site pain, bone pain, paralysis of the vocal cords, ocular toxicity, including ptosis and dysfunction of the autonomic system. High doses may cause vegetative neuropathy, including urinary retention, orthostatic hypotension, and constipation. Patients receiving vinblastine should receive opioid analgesics with caution due to the risk of additive vegetative neuropathy, which may lead to severe constipation. Severe pain in the jaw or parotid gland may occur within a few hours of the first dose of vinblastine. This is not an indication to stop treatment or change the dose; treatment with analgesics is recommended. Ototoxicity due to damage to the cranial nerve VIII is manifested by dizziness, nystagmus, vertigo and hearing impairment. Hearing impairment can be partial or total, temporary or permanent. The use of vinblastine with caution is recommended in patients receiving treatment with other potentially ototoxic drugs, such as platinum-based antineoplastics [162].
vi. Acute dyspnea and bronchospasm have occurred in cancer patients treated with vinca alkaloids and are more common in combination with mitomycin. Symptoms may include cough, dyspnoea, hypoxemia, and interstitial infiltration. Patients with pre-existing pulmonary dysfunction have an increased risk of respiratory toxicity associated with vinblastine [163].
vii. Tumor lysis syndrome can result from cell lysis by cytotoxic chemotherapy and can lead to electrolyte disturbances or acute renal failure. It is most likely with tumors with a high proliferation rate and marked tumor mass, such as leukemias, high-grade lymphomas, and myeloproliferative diseases. The risk may be increased in patients with pre-existing renal impairment, especially ureteral obstruction [164].

### Pharmacokinetic limitations

The absorption of Vinblastine in the gastrointestinal tract is unpredictable. After intravenous administration, the drug quickly disappears from the blood and is distributed to the tissues. Vinblastine crosses the blood–brain barrier to a small extent and cannot reach therapeutic concentrations in the cerebrospinal fluid. Metabolism is extensive, mainly in the liver, to des-acetylvinblastine, a much more active pharmacodynamic metabolite [158]. In addition, various genotoxicity tests have shown that vinblastine can induce chromosomal, micronuclei, and polyploid abnormalities, so Vinblastine is potentially carcinogenic [153]. The alkaloid Vincristine also crosses the blood–brain barrier with difficulty. Vincristine is metabolised by the cytochrome P450 isoenzyme in the subfamily CYP3A. This metabolic pathway may be impaired in patients with hepatic impairment or those being treated with CYP3A isoenzyme inhibitors [165].

Another limitation of alkaloids is the low water solubility and low bioavailability, as a result of which effective therapeutic concentrations are difficult to achieve in the tumor target. As a result, new strategies are needed to increase their bioavailability: structural changes, new therapeutic target transport systems, and the development of nanotechnologies [166, 167].

## Overall conclusion

Alkaloids such as Colchicine from *Colchicum autumnale*; vinblastine, vincristine, vindesine, and vinorelbine (Vinca alkaloids) from *Catharanthus roseus*; and Vincamine from *Vinca minor* are promising anticancer properties. These alkaloids through their binding to microtubulins, one of the key components of the cytoskeleton, form the tubulin-alkaloid complex. These complexes thereof inhibit cancer cell migration and metastatic potential of the cell resulting in Programmed Cell Death and apoptosis of cancer cells. These alkaloids can be used in combination with chemotherapy regimens, as they do not impart cross-resistance to commonly used DNA alkylating drugs. However, a strong spectrum of biotechnological studies on in vitro production of these compounds is required, to which plant callus suspension cultures with different biotic and abiotic elicitors in bioreactors may have the potential to be new frontiers for the production of compounds with anticancer activity. For primary and secondary chemoprevention as well as cancer management as a whole, a comprehensive research program is warranted for the discovery of new alkaloids along with their metabolic effects and molecular actions such as vinca alkaloids, to develop novel anticancer drugs followed by clinically relevant recommendations for dose and efficacy, which is often underrepresented.

## Supplementary Information

**Additional file 1.** Natural sources of alkaloids with potential anticancer effects and biotechnological studies on in-vitro culture of Alkaloids

## Data Availability

Not applicable.
