# Supplementary material for: Anticancer potential of alkaloids: a key emphasis to colchicine, vinblastine, vincristine, vindesine, vinorelbine and vincamine
Source: Cancer Cell Int. 2022 Jun 2;22:206. doi: 10.1186/s12935-022-02624-9 (PMC9161525; doi:10.1186/s12935-022-02624-9)
Supplement: Supplementary file 1 — Additional file 1. Natural sources of alkaloids with potential anticancer effects and biotechnological studies on in-vitro culture of Alkaloids [file 12935_2022_2624_MOESM1_ESM.docx]

**1. Natural sources of alkaloids with potential anticancer effects**

**1.1.** **Colchicine plant source: *Colchicum autumnale***

*Colchicum autumnale* (Family Liliaceae) is a polycarpic perennial hysteranthous geophyte and is commonly known as the autumn crocus. The whole plant is poisonous and contains alkaloid colchicine, and corm or underground bulb is a major source of colchicine (0.35%) (Brnèiæ et al., 2001).

**Synonym and common name:** *Autumn crocus*; *Crocus* (autumn); Meadow saffron

**Morphological description:** *Colchicum autumnale* is a tiny herbaceous perennial plant (10-40 cm high) that flowers in the autumn after the leaves have dropped off (Jung et al., 2011).

(i) Fruit: an oblong to ovoid green then a brown capsule containing 180-200 seeds.

(ii) Flower: The flowers are pink, purple, and white, and they come in groupings of one to six. The petal is small (3-4.5 cm) and fused below into a pale stalk-like tube (5-20 cm).

(iii) Leaves: leaves are shiny dark green. These leaves appear in the spring and usually perish before the flowers bloom (Huxley, 1992).

**Geographical distribution:** *C. autumnale* is a native plant of the South, West, and central Europe. The geographical distribution includes occurrence in Austria, Belgium, Bulgaria, Croatia, Czechia, France, Germany; Greece, Hungary, Ireland, Italy, Netherlands, Poland, Romania, Slovenia, Spain, Switzerland, Ukraine, United Kingdom.

**Habitat:** The species is found in damp meadows, open woods, and river banks. It grows moderately in nutrient-rich and deep soils with pH values of 4-8. The altitudinal range of distribution is from near sea level to 1,700 m (Chadburn, 2014).

**1.2.**  **Vinca alkaloids plant source: *Catharanthus roseus***

**Synonym and common name:** Madagascar periwinkle, Bright eyes, Cape periwinkle, Graveyard plant

**Morphological description:** It is a perennial subshrub that is woody at the base and up to 1 m tall. The detailed morphological features are as follows:

(i) Fruit: It is green in color and composed of two cylindrical follicles (2-4.5 cm long) encompassing 10-20 seeds. It is shortly hairy to glabrous and striate (Grubben and Denton, 2015).

(ii) Inflorescence: It is terminal and 1-2 flowered.

(iii) Flowers: the flower is bisexual, regular, and 5-merous. The sepals are 3-5 mm long, erect, green, and slightly fused at the base. The corolla tube is 2-3 cm cylindrical in shape and shortly hairy to the glabrous outside. The stamens are included and very short filaments, are incorporated and positioned beneath the corolla throat. The ovary is superior, with two narrowly oblong carpels and a thin, 15–23 mm long style.

(iii) Leaves: leaves are decussately opposite, simple, and entire. The leaf is 2.5–8.5 cm long and elliptical to obovate to narrowly obovate.

(iv) Stems: narrowly winged, green or red, and hairy to glabrous in appearance.

**Geographical distribution:** Madagascar is home to the *C. roseus*.

The plant has been naturalized and cultivated as an ornamental in tropical and subtropical climates after being introduced to numerous regions in Asia, Africa, America, southern Europe, and Oceania. (<http://www.hear.org/pier/>)

**Habitat:** Coastal habitats and sandy sites all along beaches, inland on riverbanks, savanna vegetation, dry waste places, roadsides, open forest, and scrubland are all examples of the general habitat. The plants normally thrive in sandy soils, but they can also grow in rocky soils.

**Trade availability:** Oncovin® and Velban®, two anticancer drugs produced from *C. roseus*, are sold for a total of US$100 million per year (Grubben and Denton, 2015).

**1.3 Vincamine plant source: Vinca minor, Vinca major, Vinca erecta, Vinca herbacea**

**1.3.1 Vinca minor**

*Vinca minor* is a member of the Apocynaceae family and comprises a large number of alkaloids like Vincamine and vincarubine.

**Synonym and common name**

The common periwinkle, Lesser Periwinkle, Periwinkle

**Morphological description**

(i) Fruits: the fruits are cylindrical pods and 3-5 cm long.

(ii) Flowers: the flower is single (1 per stem) and pinwheel-like 20-30 mm wide. The color is blue-purple, but sometimes it is white or violet. The flower blooms in late spring or early summer.
(iii) Leaves: leaves are dark green with a thick and glossy appearance. The leaves are oval-shaped, 3-9 cm long and are usually narrow at the base

(iv) Stems: the stem is 10-60 cm long evergreen slender. It is branched out to form a trailing and spreading groundcover.

**Geographical distribution**

It is native to central and southern Europe. From Portugal and France north through the Netherlands and the Baltic States, east to the Caucasus, and southwestern Asia in Turkey, the geographical spread is extensive.

**Habitat**

It grows in moist sites in forested areas and along watercourses. It is shade tolerant and extremely adaptable to many growing conditions.

**3.2.2 Vinca pubescens=Vinca major**

**Synonym and common name**

Bigleaf periwinkle, big periwinkle, greater periwinkle, large periwinkle, periwinkle vinca

**Morphological description**

It is a spreading evergreen herbaceous. The stem is dark green, hairless (to 1.5 m long) that can develop roots. The stems have a milky sap.

**Geographical distribution**

It is native to Mediterranean Europe, Asia Minor, and northern Africa. The geographical distribution includes its occurrence in Albania, France, Italy, Spain (Witt and Luke, 2017).

**Habitat**

*V. major* grows in deep shade and poor soils along roads, lawns, and forest understory.

**3.2.3 Vinca erecta**

**Morphological description**

It is a perennial, rootstock ligneous, horizontal, and covered with scales.

(i) Flowers: the flowers are large and pale azure. The calyx segments are 7.5–10 mm long and linear-lanceolate with thick oblong teeth at the base. The corolla tube is obovate, narrow, abruptly enlarged about the middle and 15-20 cm long.

(ii) Leaves: leaves are elliptical, acute, glabrous, and narrow at both ends.

(iii) Stems: the stem is erect, simple and 30–40 cm long. They can be two too many from the common rootstock.

(iv) Roots: the roots are ﬁrm, stringy

**Geographical distribution**

It is a native to Afghanistan, Kirgizstan, Tadzhikistan, and Uzbekistan.

**Habitat**

From the plains to the mid-mountain belt, it flourishes on coarse-skeletal soils, less frequently on stony-rubble, sandy elevations, and occasionally in Juniperus thickets (Bussmann et al., 2020).

**3.2.4 Vinca herbacea**

**Synonym and common name**

Herbal periwinkle

**Morphological description**

It is an herbaceous perennial vascular plant. It spreads over the ground and roots along the stems to produce clonal colonies like a trailing vine.

(i) Flowers: The flowers bloom in the spring. These are blue-violet or white and are evenly distributed throughout the stems. The calyx lobes are 4–8 mm long and narrowly triangular, with smooth, ciliate, or scabrid edges. The corolla has a deep blue colour and is 10–15 mm in diameter (Waldstein and Kitaibel, 1802).

(ii) Leaves: The leaves are large, glossy green, and have a complete margin. These are 1-5 cm long and narrowly lanceolate or narrowly elliptical. The lowermost leaves are obovate, cuneate at the base, and have minutely ciliate edges. Pedicels are shorter than the leaves that surround them (Stearn, 1978).

(iii) Stem: It is procumbent or ascending and length up to 80 cm.

**Geographical distribution**

The native range is Central & South East Europe to Western Asia. The plant can be found in Austria, Bulgaria, Germany, Hungary, Iran, Iraq, Krym, Lebanon-Syria, North Caucasus, Palestine, Romania, South European Russi, Transcaucasus, Turkey, Turkey-in-Europe, Ukraine, and Yugoslavia.

**Habitat**

Occurs mainly in steppe habitats

**2. Biotechnological studies on *in-vitro* culture of Alkaloids**

**1. Colchicine**

Apart from being medicinally used, it is also used to induce polyploidy in plant cells for producing improved varieties and cultivars. Also, within the Liliales, colchicine has been used as a chemical identifier for recognizing the Colchicaceae family (Nguyen et al., 2015).

Traditionally colchicines have been sourced for industrial use by field cultivation of various species of Colchicum and Gloriosa. However, the technique is time-consuming and costly as it is dependent on season, availability of raw seed and rhizome, limitations of cultivation practices, availability of ultra-pure colchicines (Sivakumar, 2018), which results in a hampered pharmaceutical-grade colchicines recovery. Given the importance of colchicines, the production of colchicines by plant tissue culture was first investigated in *Colchicum autumnale* suspension cultures by (Hayashi et al., 1988, Yoshida et al., 1988).

(Daradkeh et al., 2012), conducted studies on cell suspension and *in-vitro* production of Colchicines in *Colchicium hierosolymitanum* Fieb. and found a maximum concentration of 0.235 mg/g DW and 0.012 mg/g DW in callus and cell suspension. Different studies have studied the effects of precursors, polyamines, plant growth regulators, and elicitors to establish optimal culture conditions for colchicines production through different species such as *Gloriosa superb*, *Iphigenia indica*. The studies have found that all precursors (except trans-cinnamic acid) enhance colchicine content of root cultures with the treatment of p-coumaric acid+tyramine (Ghosh et al., 2002); optimal culture conditions for colchicines production are provided by treatments with elicitors such as NiSO4 and Tyr (Mukhopadhyay and Mukhopadhyay, 2008) the polyamines (Spermidine, Spermine, and Putrescine) in combination enhance multiple shoot induction and colchicine content for eg. Polyamines treated leaf sample have 0.005 mg/ml colchicine than in naturally grown leaves in the field (0.0024 mg/ml) (Sivakumar et al., 2019).

Although the colchicines production through callus development, adventitious roots induction, and other *in-vitro* technique have been attempted, a very low level of concentration could be harvested (Shibli et al., 2010, Ghosh et al., 2015, Bai and Agastian, 2013, Kumar et al., 2015, Sivakumar et al., 2003). (Finnie and Van Staden, 1994) reported Callus and roots in suspension culture have 10-25 times lower Colchicine content than normal filed plants in *Sandersonai aurantiaca* (commonly called Christmas bells; family Colchicaceae). It may be due to a lack of biosynthetic machinery for functional gene expression, as alkaloid biosynthetic pathway often occurs in various organs, with crosstalk among different pathways (Zhou and Memelink, 2016).

Apart from conventional i*n-vitro* production of Colchicine, (Yadav et al., 2013) used three Arbuscular mycorrhizal fungi (AMF) strains viz. *Glomus mosseae, Acaulospora laevis,* and mixed AMF strain for inducing acclimatization, growth, and enhancement of colchicines content in micro propagated *Gloriosa superb*a plantlets. While the *A. Laevis* gave the highest survival, higher colchicines content was found to be in combination with *A. laevis* and *G. mosseae* inoculated plants. Similarly, (Pandey et al., 2014) evaluated the effect of Arbuscular mycorrhiza (*Glomus mosseae, Glomus fasciculatum, Gigaspora margarita,* and *Gigaspora gilmorie*) and phosphate fertilizer on plant growth and production of colchicines in *Gloriosa superba*. The study found that the mycorrhization and/or P-fertilization increases colchicine concentration in *In-vitro* grown *Gloriosa superba*. Additionally, (Bai and Agastian, 2013) used *Agrobacterium rhizogenes* and found that nitrogen, ferric ions, and inoculums significantly affect colchicine production as well as hairy root growth in *Gloriosa superba*.

The microorganisms have also been used in the synthesis/modification and industrial production of Colchicine and its derivatives, which have the same therapeutic activities and industrial demand. Derivatives such as 3-demethylated colchicine (3-DMC), have been developed *in-vitro* by demethylation of colchicines with the help of a recombinant strain of E.coli with a *Bacillus megaterium* ACBTO3 strain (Dubey et al., 2010). The study demonstrated significant biotransformation of colchicines through recombinant E.coli at the bioreactor level for suitable commercialization. The derivatives such as glycoside colchicoside, 3-O-demethylglucoside, 3-demethylcolchicine, and 2-demethylcolchiine have been obtained in plant suspension culture of *Astragalus Vesicarius* (a plant which does not produce non-heterocyclic alkaloids) and *Colchicum Variegatum* culture, respectively (Zarev et al., 2019, Poulev et al., 1995). In addition to these *in-vitro* techniques, an alternative can be biorhizome production in a bioreactor. The meristematic tissue of the biorhizome produces new rhizomes and sprouts while the remaining rhizome can act for the biosynthesis of Colchicine (Sivakumar et al., 2019).

**2. Vincristine, Vindoline, Vinblastine**

(Aslam et al., 2009) investigated Vincristine production *in C. roseus* plantlets developed from *ex-vitro* and *in-vitro* somatic embryos. The research indicates that in-vitro raised tissues like calluses (embryogenic and non-embryogenic), embryogenic levels (propagated, matured, and germinated embryos), as well as somatic embryo-derived plantlets (leaf, root, and whole plant) could accumulate higher levels of Vincristine than *ex vitro* developed plantlets and that Vincristine yield was tissue and age-dependent. Consequently,(Kalidass et al., 2009)studied and established the effect of auxin and cytokinin in various combinations (0.5 µM to 3.0 µM) on vincristine production in callus cultures of *Catharanthus roseus*. Similarly, (Mekky et al., 2018) could successfully increase the biosynthesis of vincristine and vinblastine in callus cultures of *Catharanthus roseus* by culturing Vinca leaves on MS medium supplemented with Kin/IAA. The study could detect vincristine significantly higher than wild plants and 3.39 fold increase in vinblastine concentration.(Guillon et al., 2008), as an alternate and promising biotechnological process for in vitro production of Vinblastine cultured transgenic, *C. roseus* hairy root clones supplemented with MeJA treatment and found that Highly differentiated hairy root cultures were able to produce Vinblastine precursors as found in the natural roots of *C. roseus*.

The effect of MeJA coupled with abiotic elicitors on the production of important medicinal alkaloids is also investigated by (Paeizi et al., 2018). In micro-propagated shoots of C. roseus, the impacts of methyl jasmonate and silver nitrate on the synthesis of vincristine, vinblastine, ajmalicine, vindoline, and catharanthine were examined individually and synergistically. The study found that the maximum yield of mentioned alkaloids was under 100 µM methyl jasmonate in combination with 100 µM of AgNO after seven days. The MeJA and Ag +elicitors upregulated TIA pathway biosynthesis enzymes (e.g. STR, GS, DAT, and CrPRX1) through activation of transcription factors such as ORCA3 with possible crosstalk between silver nitrate and methyl jasmonate signaling pathways, according to the study. Many studies have looked at how methyl jasmonate, as well as abiotic stimuli such as low light, UV-B, drought, and salt, as well as ß cyclodextrin, ethylene, and chromium, affect the elicitation of alkaloids in *C. roseus*. (Liu et al., 2011, Binder et al., 2009, Jaleel et al., 2007, Jaleel et al., 2008, Osman et al., 2007, Wang et al., 2016, Rai et al., 2014). In continuation, studies have found that Catharanthus alkaloids are growth-specific and can be influenced by abiotic elicitors such as sodium chloride and artemisinic acid.(Fatima et al., 2015), has found that Vinblastine and Vincristine content was maximum in regenerated leaves on 25mM NaCl amended medium as compared to *in-vitro* raised shoots. (Liu et al., 2014) used Artemisinic acid elicitation to increase Vindoline and Vinblastine synthesis in suspension-cultured *Catharanthus roseus* cells. According to the findings, vindoline and vinblastine concentrations were raised six-fold and two-fold, respectively. Artemisinic acid was also found to be able to up-regulate the transcription of the TIA pathway enzymes Tryptophan Decarboxylase (TDC), Geraniol 10-hydroxylase (G10H), Tabersonine 16-hydroxylase (T16H), and Deacetoxy vindoline 4-hydroxylase (D4H). Using different elicitors of hydroxylase, peroxidase, acetyltransferase, and inhibitors of oxygenase to a *Catharanthus roseus* cell culture media, (Guo et al., 2013) investigated the modulation of vinblastine production in cell suspension cultures of *Catharanthus roseus*. The study found that the Vindoline concentration could be enhanced with incorporating 30 lg/L acetyl CoA into the media. The study also found that most vinblastine may be effectively enhanced with the help of 5 mg/L acetyl CoA, 20 lg/L hydrogen peroxide, 0.5lmol/L benzotriazole, 100 mg/L tryptophan, 100 mg/L loganin, and 30 mg/L cerium chloride. In the study, Simultaneous variations in the activity of Cytochrome P-450 monooxygenases, Deacetyl vindoline-O-acetyltransferase, and Peroxidase enzymes were observed under experimental conditions, indicating that these enzymes are strongly connected to vinblastine production. Although the Vinblastine was produced in all treatment groups, acetyl CoA and tryptophan were the most important factors in vinblastine biosynthesis, according to the research.

To enhance the concentration of Vinblastin and Vincristine by using biotic elicitors in *C. roseus* callus, (Pliankong et al., 2018) used chitosan from shrimp shells was a successful biotic elicitor to enhance Vinblastin and Vincristine in *Catharanthus roseus* cell cultures. Similarly, Study conducted by (Birat et al., 2021) used endophyte Alternaria sesame fungal extract to augment the concentration of Vincristine in *C. roseus* callus *in-vitro*. The study established that the above treatment enhanced the Vincristine content by 21.717% after 105 days *in-vitro* culture.

An array of studies have focussed to harvest Vinblastine from fungal strains such as *Alternaria alternate* isolated from *Melissa officinalis* (El‐Sayed, 2021), *Fusarium oxysporum* (Kumar et al., 2013), *Talaromyces radicus* (Palem et al., 2015), and *Curvularia verruculosa* from *Catharanthus roseus* (Parthasarathy et al., 2020). (Ramezani et al., 2018) investigated the effects of Priformospora and Trichoderma fungal extracts applied to *C. roseus* cell suspensions at various concentrations and time durations on vinblastine and vincristine production. The results of the study showed that amount of vinblastine was increased however vincristine amount decreased with treatment. Overall efficacy of these preparations on the expression profiles of vindoline 4-O-acetyltransferase (DAT) and Desaccetoxyvindoline-4-Hydroxylase (D4H) genes was considerable, according to the study, and their expression levels were enhanced in mRNA level. (Maqsood and Abdul, 2017) used yeast extract as a biotic elicitor in *C. roseus* protoplast-derived tissues and plantlets for Vinblastine and Vincristine. The study established that the treatment of yeast extract significantly increased Vinblastine and Vincristine content. Nonetheless, the activity of yeast extract has been linked to the stimulation of the TIA biosynthetic genes STR and TDC, which encode Strictosidine synthase and tryptophan decarboxylase, respectively, in alkaloid production (Pauw et al., 2004.)

Apart from using various in-vitro approaches using growth hormones, abiotic and biotic elicitors amended cultures, Studies have also focused on metabolic engineering approaches to enhance the content of Vinblastine and Vincristine. Accordingly, (Sharma et al., 2018) investigated the metabolic engineering of vinblastine in *C. roseus* and found that overexpressing two upstream TIA pathway genes, tryptophan decarboxylase (CrTDC) and Strictosidine synthase, increased the metabolic flux of the TIA system (CrSTR). The study developed plant transgenic using Agrobacterium tumefaciens LBA1119 strain having CrTDC and CrSTR gene. A twofold increase in total alkaloid and nine-fold increase in Vindoline and Catharanthine and a fivefold increase in vinblastine content were observed in the transgenic lines. Later (Sharma et al., 2019) used multiple shoot cultures of *Catharanthus roseus* along with callus cultures subjected to abiotic elicitors and TIAs (Terpenoid Indole Alkaloids) pathway precursors such as tryptamine and tryptophan to study the complexity and production of Vinblastine. The study established that multiple shoot cultures can provide cellular complexity for completing the TIA pathway, therefore solving the problem associated with Cell suspension, hairy roots, and callus cultures for failing to complete the TIA pathway. It is also established that Vinblastine can also be made semi-synthetically by combining Catharanthine with Vindoline derived from cultured plants (Jacobs et al., 2004)

**3.6 Potential biotechnological studies on *in-vitro* culture**

Tissue culture, cell suspensions, and hairy roots are being used to make these precious medications without having to cut down entire plants that have lived for decades. The application of elicitors in plants has been proven to boost the synthesis of some secondary metabolites. (Molchan et al., 2012) monitored L-tryptophan decarboxylase (TDC) and tryptamine accumulation in *Vinca minor* leaf tissue callus cultures. In the study, the Callus of V. minor demonstrated signiﬁcant TDC activity and tryptamine content which can potentially be used for commercial production of tryptamine derivatives, such as Vincamine. (Verma et al., 2012) found that in-vitro selection and maintenance of three stable variant shoot lines of V. minor (V10, V20, and V30) with sensitivity threshold values of 10, 20, and 30 mg/l 5-methyltryptophan (5-MT; analogue of tryptophan), respectively, on shoot multiplication medium supplemented with BA and NAA could result in a maximum 16-fold increase in Vincamine production. Thus highlighting the importance of *in-vitro* production for Vincamine content.

(Xu et al., 2020) improved the production of Vincamine in endophytic fungus (*Tausonia pullulans* strain) isolated from *Vinca minor* by optimizing and subsequently via the inactivated protoplast fusion method. As compared to the parent strain, two fusants strains were able to show 162.24 and 221.16%, respectively higher content of Vincamine. Utilizing mutagens to immobilize protoplasts resulted in more fusants and high Vincamine synthesis strains, according to the study. A study conducted by (Verma et al., 2014) used four endophytic fungi namely *Chaetomium globosum; Aspergillus niveoglaucus; Paecilomyces lilacinus,* and *Trichoderma harzianum* culture filtrate on cell suspension of hairy root clone of *Vinca minor* at flask culture and bioreactor level. The culture ﬁltrate of *T. harzianum* enhanced the Vincamine content in hairy roots and cell suspensions. (Verma et al., 2014) investigated the effect of elicitors in Vincamine production, including hydroxylase and acetyltransferase elicitors, as well as precursor availability and cyclooxygenase inhibitors. The authors could fortify the hairy root clone culture with the mentioned factors. Once 20-day-old hairy roots were reinforced with Secologanin, tryptophan, naproxen, hydrogen peroxide, and acetic anhydride, the greatest flow towards Vincamine synthesis was observed. The activities of tryptophan decarboxylase and Strictosidine synthase were found to be 2- and 3-fold higher in the study, respectively.

**References**

AFSAHUL, K. M. & ANJUM, F. 2020. Suranjan (Colchicum autumnale L. and merendra persica): Great resolvent herbs of Unani system of medicine-a review. *Int. J. Unani Integr. Med.,* 4**,** 7-11.

AGARWAL, S., JACOB, S., CHETTRI, N., BISOYI, S., TAZEEN, A., VEDAMURTHY, A., KRISHNA, V. & HOSKERI, H. 2011. Evaluation of in-vitro anthelminthic activity of Catharanthus roseus extract. *Int J Pharm Sci Drug Res,* 3**,** 211-3.

AL-RASHED, S., BAKER, A., AHMAD, S. S., SYED, A., BAHKALI, A. H., ELGORBAN, A. M. & KHAN, M. S. 2021. Vincamine, a safe natural alkaloid, represents a novel anticancer agent. *Bioorganic Chemistry,* 107**,** 104626.

ALMAGRO, L., FERNÁNDEZ-PÉREZ, F. & PEDREÑO, M. A. 2015. Indole alkaloids from Catharanthus roseus: bioproduction and their effect on human health. *Molecules,* 20**,** 2973-3000.

ALTINOZ, M. A., OZPINAR, A., ALTURFAN, E. E. & ELMACI, I. 2018. Vinorelbine’s anti-tumor actions may depend on the mitotic apoptosis, autophagy and inflammation: hypotheses with implications for chemo-immunotherapy of advanced cancers and pediatric gliomas. *Journal of Chemotherapy,* 30**,** 203-212.

AMERI, A., SHAHRAD, B., FAZLALIZADEH, A., MADANI, H., MOUSAVIZADEH, A. & MOGHADAM, S. 2013. Vinorelbine and Docetaxel Combination as the First Line Treatment in Patients with Metastatic Breast Cancer: Results of a Multi-centric Phase II Trial in Iran. *Reports of Radiotherapy and Oncology,* 1**,** 51-7.

ANSARI, A., SALEEM, S. & KALAM, A. 2019. Management of Wajaʹ al-Rakba (knee Osteoarthritis) by TakmīdHārRatab (hot and moist fomentation) and Habb-i-Sūranjān: A case study. *International Journal of AYUSH Case Reports,* 3**,** 60-68.

ARORA, R., MALHOTRA, P., MATHUR, A. K., MATHUR, A., GOVIL, C. & AHUJA, P. 2010. Anticancer alkaloids of Catharanthus roseus: transition from traditional to modern medicine. *Herbal Medicine: A Cancer Chemopreventive and Therapeutic Perspective. Jaypee Brothers Medical Publishers Pvt. Ltd, New Delhi, India***,** 292-310.

ASLAM, J., KHAN, S. H., SIDDIQUI, Z. H., FATIMA, Z., MAQSOOD, M., BHAT, M. A., NASIM, S. A., ILAH, A., AHMAD, I. Z. & KHAN, S. A. 2010. Catharanthus roseus (L.) G. Don. An important drug: it’s applications and production. *Pharmacie Globale (IJCP),* 4**,** 1-16.

ASTANI, A., REICHLING, J. & SCHNITZLER, P. 2012. <i>Melissa officinalis</i> Extract Inhibits Attachment of Herpes Simplex Virus in vitro. *Chemotherapy,* 58**,** 70-77.

BAIRY, K., SANATH, S., JAGETIA, G., SOMAYAJI, S., VIDYASAGAR, M. & BALIGA, M. 2003. Evaluation of intraperitoneal vincristine in malignant peritoneal effusion. *Indian journal of physiology and pharmacology,* 47**,** 270-278.

BAKAR-ATEŞ, F., ÖZMEN, N., KAYA-SEZGINER, E. & KURT, E. E. 2018. Effects of colchicine on cell cycle arrest and MMP-2 mRNA expression in MCF-7 breast adenocarcinoma cells. *Turk. Hij. Den. Biyol. Derg,* 75**,** 239-244.

BARBIER, P., TSVETKOV, P. O., BREUZARD, G. & DEVRED, F. 2014. Deciphering the molecular mechanisms of anti-tubulin plant derived drugs. *Phytochemistry reviews,* 13**,** 157-169.

BARRALES-CUREÑO, H. J. 2015. Pharmacological applications and in vitro biotechnological production of anticancer alkaloids of Catharanthus roseus. *Biotecnol Apl,* 32**,** 1101-10.

BATES, D. & EASTMAN, A. 2017. Microtubule destabilising agents: far more than just antimitotic anticancer drugs. *British journal of clinical pharmacology,* 83**,** 255-268.

BECK, W. T., CASS, C. E. & HOUGHTON, P. J. 2000. Microtubule-targeting anticancer drugs derived from plants and microbes: vinca alkaloids, taxanes, and epothilones. *Holland-Frei Cancer Medicine. 5th edition.* BC Decker.

BEDIKIAN, A. Y., VARDELEON, A., SMITH, T., CAMPBELL, S. & NAMDARI, R. 2006. Pharmacokinetics and urinary excretion of vincristine sulfate liposomes injection in metastatic melanoma patients. *The Journal of Clinical Pharmacology,* 46**,** 727-737.

BENNOUNA, J., DELORD, J.-P., CAMPONE, M. & NGUYEN, L. 2008. Vinflunine: a new microtubule inhibitor agent. *Clinical cancer research,* 14**,** 1625-1632.

BHANDARI, P. & MUKERJI, B. 1959. Lochnera rosea Linn Reichb. *Gauhati Ayurvedic Coll Mag,* 8**,** 1-4.

BHUTKAR, M. & BHISE, S. 2011. Studies on Antioxidant Properties of Catharanthus rosea and Catharanthus alba. *Journal of Current Pharma Research,* 1**,** 337.

BLAJESKI, A. L., PHAN, V. A., KOTTKE, T. J. & KAUFMANN, S. H. 2002. G 1 and G 2 cell-cycle arrest following microtubule depolymerization in human breast cancer cells. *The Journal of clinical investigation,* 110**,** 91-99.

BORODY, T. J., RAMRAKHA, S., SAXON, J. & WETTSTEIN, A. 2018. Gastric and colonic formulations and methods for making and using them. Google Patents.

BRANDAO, M., BOTELHO, M. & KRETTLI, E. 1985. Antimalarial experimental chemotherapy using natural products. I. A more rational approach? *Ciência e Cultura,* 37**,** 1152-1163.

BRVAR, M., PLOJ, T., KOZELJ, G., MOZINA, M., NOC, M. & BUNC, M. 2004. Case report: fatal poisoning with Colchicum autumnale. *Critical Care,* 8**,** 1-4.

BUGA, A. M., DOCEA, A. O., ALBU, C., MALIN, R. D., BRANISTEANU, D. E., IANOSI, G., IANOSI, S. L., IORDACHE, A. & CALINA, D. 2019. Molecular and cellular stratagem of brain metastases associated with melanoma. *Oncol Lett,* 17**,** 4170-4175.

CAPPELLANO, A. M., PETRILLI, A. S., DA SILVA, N. S., SILVA, F. A., PAIVA, P. M., CAVALHEIRO, S. & BOUFFET, E. 2015. Single agent vinorelbine in pediatric patients with progressive optic pathway glioma. *Journal of neuro-oncology,* 121**,** 405-412.

CHAO, M.-W., LAI, M.-J., LIOU, J.-P., CHANG, Y.-L., WANG, J.-C., PAN, S.-L. & TENG, C.-M. 2015. The synergic effect of vincristine and vorinostat in leukemia in vitro and in vivo. *Journal of hematology & oncology,* 8**,** 1-15.

CHARPENTIER, M. S., WHIPPLE, R. A., VITOLO, M. I., BOGGS, A. E., SLOVIC, J., THOMPSON, K. N., BHANDARY, L. & MARTIN, S. S. 2014. Curcumin targets breast cancer stem–like cells with microtentacles that persist in mammospheres and promote reattachment. *Cancer research,* 74**,** 1250-1260.

CHATTOPADHYAY, R. 1999. A comparative evaluation of some blood sugar lowering agents of plant origin. *Journal of ethnopharmacology,* 67**,** 367-372.

CHATTOPADHYAY, R., SARKAR, S., GANGULY, S., BANERJEE, R. & BASU, T. 1991. Hypoglycemic and antihyperglycemic effect of leaves of Vinca rosea linn. *Indian Journal of Physiology and Pharmacology,* 35**,** 145-151.

CHEN, X.-M., LIU, J., WANG, T. & SHANG, J. 2012. Colchicine-induced apoptosis in human normal liver L-02 cells by mitochondrial mediated pathways. *Toxicology in vitro,* 26**,** 649-655.

CHENG, Z., LU, X. & FENG, B. 2020. A review of research progress of antitumor drugs based on tubulin targets. *TRANSLATIONAL CANCER RESEARCH,* 9**,** 4020-4027.

CHO, J. H., JOO, Y. H., SHIN, E. Y., PARK, E. J. & KIM, M. S. 2017. Anticancer effects of colchicine on hypopharyngeal cancer. *Anticancer research,* 37**,** 6269-6280.

DOCEA, A. O., MITRUT, P., GRIGORE, D., PIRICI, D., CALINA, D. C. & GOFITA, E. 2012. Immunohistochemical expression of TGF beta (TGF-beta), TGF beta receptor 1 (TGFBR1), and Ki67 in intestinal variant of gastric adenocarcinomas. *Romanian Journal of Morphology and Embryology,* 53**,** 683-692.

DUFLOS, A., KRUCZYNSKI, A. & BARRET, J.-M. 2002. Novel aspects of natural and modified vinca alkaloids. *Current Medicinal Chemistry-Anti-Cancer Agents,* 2**,** 55-70.

FARNSWORTH, N. Plants and modern medicine: where science and folklore Meets. World Health Forum, 1985. 76-80.

FERRERES, F., PEREIRA, D. M., VALENTÃO, P. C., ANDRADE, P. B., SEABRA, R. M. & SOTTOMAYOR, M. 2008. New phenolic compounds and antioxidant potential of Catharanthus roseus. *Journal of Agricultural and Food Chemistry,* 56**,** 9967-9974.

FINKELSTEIN, Y., AKS, S. E., HUTSON, J. R., JUURLINK, D. N., NGUYEN, P., DUBNOV-RAZ, G., POLLAK, U., KOREN, G. & BENTUR, Y. 2010. Colchicine poisoning: the dark side of an ancient drug. *Clinical toxicology,* 48**,** 407-414.

FLEURY, C., MIGNOTTE, B. & VAYSSIÈRE, J.-L. 2002. Mitochondrial reactive oxygen species in cell death signaling. *Biochimie,* 84**,** 131-141.

FOYE, W. O. 1995. *Cancer chemotherapeutic agents*, Amer Chemical Society.

GAJALAKSHMI, S., VIJAYALAKSHMI, S. & DEVI, R. V. 2013. Pharmacological activities of Catharanthus roseus: a perspective review. *International Journal of Pharma and Bio Sciences,* 4**,** 431-439.

GANGULY, A., YANG, H., ZHANG, H., CABRAL, F. & PATEL, K. D. 2013. Microtubule dynamics control tail retraction in migrating vascular endothelial cells. *Molecular cancer therapeutics,* 12**,** 2837-2846.

GOA, K. L. & FAULDS, D. 1994. Vinorelbine. *Drugs & aging,* 5**,** 200-234.

GRATTAGLIANO, I., BONFRATE, L., RUGGIERO, V., SCACCIANOCE, G., PALASCIANO, G. & PORTINCASA, P. 2014. Novel therapeutics for the treatment of familial Mediterranean fever: from colchicine to biologics. *Clinical Pharmacology & Therapeutics,* 95**,** 89-97.

GREGORY, R. & SMITH, I. 2000. Vinorelbine–a clinical review. *British journal of cancer,* 82**,** 1907-1913.

GROTH-PEDERSEN, L., OSTENFELD, M. S., HØYER-HANSEN, M., NYLANDSTED, J. & JÄÄTTELÄ, M. 2007. Vincristine induces dramatic lysosomal changes and sensitizes cancer cells to lysosome-destabilizing siramesine. *Cancer research,* 67**,** 2217-2225.

HABLI, Z., TOUMIEH, G., FATFAT, M., RAHAL, O. N. & GALI-MUHTASIB, H. 2017. Emerging cytotoxic alkaloids in the battle against cancer: Overview of molecular mechanisms. *Molecules,* 22**,** 250.

HALDAR, S., JENA, N. & CROCE, C. M. 1995. Inactivation of Bcl-2 by phosphorylation. *Proceedings of the National Academy of Sciences,* 92**,** 4507-4511.

HASSAN, K. A., BRENDA, A. T., PATRICK, V. & PATRICK, O. E. 2011. In vivo antidiarrheal activity of the ethanolic leaf extract of Catharanthus roseus Linn.(Apocyanaceae) in Wistar rats. *African Journal of Pharmacy and Pharmacology,* 5**,** 1797-1800.

HEINRICH, M., APPENDINO, G., EFFERTH, T., FÜRST, R., IZZO, A. A., KAYSER, O., PEZZUTO, J. M. & VILJOEN, A. 2020. Best practice in research – Overcoming common challenges in phytopharmacological research. *Journal of Ethnopharmacology,* 246**,** 112230.

HOSSAIN, R., QUISPE, C., HERRERA-BRAVO, J., ISLAM, M. S., SARKAR, C., ISLAM, M. T., MARTORELL, M., CRUZ-MARTINS, N., AL-HARRASI, A., AL-RAWAHI, A., SHARIFI-RAD, J., IBRAYEVA, M., DAŞTAN, S. D., ALSHEHRI, M. M., CALINA, D. & CHO, W. C. 2021. <i>Lasia spinosa</i> Chemical Composition and Therapeutic Potential: A Literature-Based Review. *Oxidative Medicine and Cellular Longevity,* 2021**,** 1602437.

HSU, F. L. & CHENG, J. T. 1992. Investigation in rats of the antihyperglycemic effect of plant extracts used in taiwan for the treatment of diabetes mellitus. *Phytotherapy Research,* 6**,** 108-111.

HUANG, Z., XU, Y. & PENG, W. 2015. Colchicine induces apoptosis in HT‑29 human colon cancer cells via the AKT and c-Jun N-terminal kinase signaling pathways. *Molecular medicine reports,* 12**,** 5939-5944.

ISLAM, M. T., QUISPE, C., EL-KERSH, D. M., SHILL, M. C., BHARDWAJ, K., BHARDWAJ, P., SHARIFI-RAD, J., MARTORELL, M., HOSSAIN, R., AL-HARRASI, A., AL-RAWAHI, A., BUTNARIU, M., ROTARIU, L. S., SULERIA, H. A. R., TAHERI, Y., DOCEA, A. O., CALINA, D. & CHO, W. C. 2021. A Literature-Based Update on <i>Benincasa hispida</i> (Thunb.) Cogn.: Traditional Uses, Nutraceutical, and Phytopharmacological Profiles. *Oxidative Medicine and Cellular Longevity,* 2021**,** 6349041.

JAIN, D., CHAUDHARY, P., VARSHNEY, N., BIN RAZZAK, K. S., VERMA, D., KHAN ZAHRA, T. R., JANMEDA, P., SHARIFI-RAD, J., DAŞTAN, S. D., MAHMUD, S., DOCEA, A. O. & CALINA, D. 2021. Tobacco Smoking and Liver <i>Cancer</i> Risk: Potential Avenues for Carcinogenesis. *Journal of Oncology,* 2021**,** 5905357.

JEWKES, J., HARPER, P., TOBIAS, J., GEDDES, D., SOUHAMI, R. & SPIRO, S. 1983. Comparison of vincristine and vindesine in the treatment of inoperable non-small cell bronchial carcinoma. *Cancer treatment reports,* 67**,** 1119-1121.

JOEL, S. 1995. The comparative clinical pharmacology of vincristine and vindesine: Does vindesine offer any advantage in clinical use? *Cancer treatment reviews,* 21**,** 513-525.

JOHNSON, I. S., ARMSTRONG, J. G., GORMAN, M. & BURNETT, J. P. 1963. The vinca alkaloids: a new class of oncolytic agents. *Cancer research,* 23**,** 1390-1427.

JORDAN, M. 2002. Mechanism of action of antitumor drugs that interact with microtubules and tubulin. *Current Medicinal Chemistry-Anti-Cancer Agents,* 2**,** 1-17.

JORDAN, M. A., THROWER, D. & WILSON, L. 1992. Effects of vinblastine, podophyllotoxin and nocodazole on mitotic spindles. Implications for the role of microtubule dynamics in mitosis. *Journal of cell science,* 102**,** 401-416.

KARAHALIL, B., YARDıM-AKAYDIN, S. & NACAK BAYTAS, S. 2019. An overview of microtubule targeting agents for cancer therapy. *Arhiv za higijenu rada i toksikologiju,* 70**,** 160-172.

KEGLEVICH, P., HAZAI, L., KALAUS, G. & SZÁNTAY, C. 2012. Modifications on the basic skeletons of vinblastine and vincristine. *Molecules,* 17**,** 5893-5914.

KHANAVI, M., POURMOSLEMI, S., FARAHANIKIA, B., HADJIAKHOONDI, A. & OSTAD, S. N. 2010. Cytotoxicity of Vinca minor. *Pharmaceutical biology,* 48**,** 96-100.

KINGSTON, D. G. I. 2009. Tubulin-Interactive Natural Products as Anticancer Agents. *Journal of Natural Products,* 72**,** 507-515.

KINTZ, P., JAMEY, C., TRACQUI, A. & MANGIN, P. 1997. Colchicine poisoning: report of a fatal case and presentation of an HPLC procedure for body fluid and tissue analyses. *Journal of analytical toxicology,* 21**,** 70-72.

KOKLESOVA, L., LISKOVA, A., SAMEC, M., QARADAKHI, T., ZULLI, A., SMEJKAL, K., KAJO, K., JAKUBIKOVA, J., BEHZADI, P. & PEC, M. 2020. Genoprotective activities of plant natural substances in cancer and chemopreventive strategies in the context of 3P medicine. *EPMA Journal,* 11**,** 261-287.

KUMAR, A. 2016. Vincristine and vinblastine: a review. *IJMPS,* 6**,** 23-30.

KUMAR, S., SINGH, B. & SINGH, R. 2021. Catharanthus roseus (L.) G. Don: A review of its ethnobotany, phytochemistry, ethnopharmacology and toxicities. *Journal of Ethnopharmacology***,** 114647.

KUREK, J. 2018. Cytotoxic colchicine alkaloids: from plants to drugs. *Cytotoxicity,* 6**,** 10.5772.

LEUNG, Y. Y., HUI, L. L. Y. & KRAUS, V. B. Colchicine—update on mechanisms of action and therapeutic uses. Seminars in arthritis and rheumatism, 2015. Elsevier, 341-350.

LIN, Z.-Y., KUO, C.-H., WU, D.-C. & CHUANG, W.-L. 2016. Anticancer effects of clinically acceptable colchicine concentrations on human gastric cancer cell lines. *The Kaohsiung journal of medical sciences,* 32**,** 68-73.

MANDHARE, A. & BANERJEE, P. 2016. Therapeutic use of colchicine and its derivatives: a patent review. *Expert opinion on therapeutic patents,* 26**,** 1157-1174.

MARTINO, E., CASAMASSIMA, G., CASTIGLIONE, S., CELLUPICA, E., PANTALONE, S., PAPAGNI, F., RUI, M., SICILIANO, A. M. & COLLINA, S. 2018. Vinca alkaloids and analogues as anti-cancer agents: Looking back, peering ahead. *Bioorganic & medicinal chemistry letters,* 28**,** 2816-2826.

MEYERS, M. A. 2007. *Happy accidents: Serendipity in modern medical breakthroughs*, Arcade Publishing.

MISHRA, J. N. & VERMA, N. K. 2017. A brief study on Catharanthus roseus: A review. *Intern J Res Pharmacy Pharmaceut Sci,* 2**,** 20-23.

MITRUT, P., DOCEA, A. O., KAMAL, A. M., MITRUT, R., CALINA, D., GOFITA, E., PADUREANU, V., GRUIA, C. & STREBA, L. 2016. *Colorectal Cancer and Inflammatory Bowel Disease*.

MOUDI, M., GO, R., YIEN, C. Y. S. & NAZRE, M. 2013. Vinca alkaloids. *International journal of preventive medicine,* 4**,** 1231.

NAYAK, B., ANDERSON, M. & PEREIRA, L. P. 2007. Evaluation of wound-healing potential of Catharanthus roseus leaf extract in rats. *Fitoterapia,* 78**,** 540-544.

NETT, R. S., LAU, W. & SATTELY, E. S. 2020. Discovery and engineering of colchicine alkaloid biosynthesis. *Nature,* 584**,** 148-153.

NGUYWEN, V. D. List of simple drugs and medicinal plants of value in Vietnam. Proc Seminar of the use of Medicinal Plants in Healthcare. Tokyo, 1977. 13-17.

NIEL, E. & SCHERRMANN, J.-M. 2006. Colchicine today. *Joint Bone Spine,* 73**,** 672-678.

NIELSEN, D., DOMBERNOWSKY, P., SKOVSGAARD, T., JENSEN, J., ANDERSEN, E., ENGELHOLM, S. & HANSEN, M. 1990. Epirubicin or epirubicin and vindesine in advanced breast cancer. A phase III study. *Annals of Oncology,* 1**,** 275-280.

NISAR, A., MAMAT, A. S., HATIM, M. I., ASLAM, M. S. & SYARHABIL, M. 2016. An updated review on Catharanthus roseus: phytochemical and pharmacological analysis. *Indian Research Journal of Pharmacy and Science,* 3**,** 631-653.

NOBLE, R. L., BEER, C. & CUTTS, J. 1958. Role of chance observations in chemotherapy: Vinca rosea. *Annals of the New York Academy of Sciences,* 76**,** 882-894.

NOSÁLOVÁ, V., MACHOVA, J. & BABULOVÁ, A. 1993. Protective action of vinpocetine against experimentally induced gastric damage in rats. *Arzneimittel-forschung,* 43**,** 981-985.

NUKI, G. 2008. Colchicine: its mechanism of action and efficacy in crystal-induced inflammation. *Current rheumatology reports,* 10**,** 218-227.

PATEL, Y., VADGAMA, V., BAXI, S. & TRIPATHI, C. B. 2011. Evaluation of hypolipidemic activity of leaf juice of Catharanthus roseus (Linn.) G. Donn. in guinea pigs. *Acta Pol Pharm,* 68**,** 927-935.

PATIL, P. J. & GHOSH, J. S. 2010. Antimicrobial activity of Catharanthus roseus–a detailed study. *British Journal of Pharmacology and Toxicology,* 1**,** 40-44.

PEREIRA, D. M., FERRERES, F., OLIVEIRA, J., VALENTÃO, P., ANDRADE, P. B. & SOTTOMAYOR, M. 2009. Targeted metabolite analysis of Catharanthus roseus and its biological potential. *Food and chemical toxicology,* 47**,** 1349-1354.

PEREIRA, D. M., FERRERES, F., OLIVEIRA, J. M., GASPAR, L., FARIA, J., VALENTÃO, P., SOTTOMAYOR, M. & ANDRADE, P. B. 2010. Pharmacological effects of Catharanthus roseus root alkaloids in acetylcholinesterase inhibition and cholinergic neurotransmission. *Phytomedicine,* 17**,** 646-652.

PEREZ, E. A. 2009. Microtubule inhibitors: Differentiating tubulin-inhibiting agents based on mechanisms of action, clinical activity, and resistance. *Molecular cancer therapeutics,* 8**,** 2086-2095.

PERNOT, B., GYAN, E., MAILLOT, F., HODGES, P., ERTAULT, M. & FERREIRA-MALDENT, N. 2018. Lymphomas diagnosed in an internal medicine department compared to lymphomas diagnosed in other departments: Clinical and outcome differences. *Medicine,* 97.

PHAM, H. N. T., VUONG, Q. V., BOWYER, M. C. & SCARLETT, C. J. 2020. Phytochemicals derived from Catharanthus roseus and their health benefits. *Technologies,* 8**,** 80.

PILLAY, P., NAIR, C. & SANTI KUMARI, T. 1959. Lochnera rosea as a potential source of hypotensive and other remedies. *Bulletin of Research Institute of the University of Kerala,* 1**,** 51-54.

PLANTLIST, T. Available: http://www.theplantlist.org/ [Accessed 2021].

RAJPUT, M. S., NAIR, V., CHAUHAN, A., JAWANJAL, H. & DANGE, V. 2011. Evaluation of antidiarrheal activity of aerial parts of Vinca major in experimental animals. *Middle-East Journal of Scientific Research,* 7**,** 784-788.

ROBINSON, K. P. & CHAN, J. J. 2018. Colchicine in dermatology: A review. *Australasian Journal of Dermatology,* 59**,** 278-285.

ROBINSON, P. C., TERKELTAUB, R., PILLINGER, M. H., SHAH, B., KARALIS, V., KARATZA, E., LIEW, D., IMAZIO, M., CORNEL, J. H. & THOMPSON, P. L. 2021. Consensus statement regarding the efficacy and safety of long-term low-dose colchicine in gout and cardiovascular disease. *The American journal of medicine*.

ROUBILLE, F., MERLET, N., BUSSEUIL, D., FERRON, M., SHI, Y., MIHALACHE-AVRAM, T., MECTEAU, M., BRAND, G., RIVAS, D. & COSSETTE, M. 2021. Colchicine reduces atherosclerotic plaque vulnerability in rabbits. *Atherosclerosis Plus*.

SALEHI, B., PRAKASH MISHRA, A., NIGAM, M., KARAZHAN, N., SHUKLA, I., KIEŁTYKA-DADASIEWICZ, A., SAWICKA, B., GŁOWACKA, A., ABU-DARWISH, M. S., HUSSEIN TARAWNEH, A., GADETSKAYA, A. V., CABRAL, C., SALGUEIRO, L., VICTORIANO, M., MARTORELL, M., DOCEA, A. O., ABDOLSHAHI, A., CALINA, D. & SHARIFI-RAD, J. 2021. Ficus plants: State of the art from a phytochemical, pharmacological, and toxicological perspective. *Phytotherapy Research,* 35**,** 1187-1217.

SANI, T. A., MOHAMMADPOUR, E., MOHAMMADI, A., MEMARIANI, T., YAZDI, M. V., REZAEE, R., CALINA, D., DOCEA, A. O., GOUMENOU, M., ETEMAD, L. & SHAHSAVAND, S. 2017. CYTOTOXIC AND APOPTOGENIC PROPERTIES OF DRACOCEPHALUM KOTSCHYI AERIAL PART DIFFERENT FRACTIONS ON CALU-6 AND MEHR-80 LUNG CANCER CELL LINES. *Farmacia,* 65**,** 189-199.

SAYEED, M., JESMIN, M. H., SARKER, T., RAHMAN, M. & ALAM, M. 2014. Antitumor Activity of Leaf Extracts of Catharanthus roseus (L.) G. Don.

SCHILLER, G. J., DAMON, L. E., COUTRE, S. E., HSU, P., BHAT, G. & DOUER, D. 2018. High-dose vincristine sulfate liposome injection, for advanced, relapsed, or refractory philadelphia chromosome-negative acute lymphoblastic leukemia in an adolescent and young adult subgroup of a phase 2 clinical trial. *Journal of adolescent and young adult oncology,* 7**,** 546-552.

SEKAR, P. 1996. Vedic clues to memory enhancer. *The Hindu, March,* 21.

SERTEL, S., FU, Y., ZU, Y., REBACZ, B., KONKIMALLA, B., PLINKERT, P. K., KRÄMER, A., GERTSCH, J. & EFFERTH, T. 2011. Molecular docking and pharmacogenomics of vinca alkaloids and their monomeric precursors, vindoline and catharanthine. *Biochemical pharmacology,* 81**,** 723-735.

SHARIFI-RAD, J., BAHUKHANDI, A., DHYANI, P., SATI, P., CAPANOGLU, E., DOCEA, A. O., AL-HARRASI, A., DEY, A. & CALINA, D. 2021a. Therapeutic Potential of Neoechinulins and Their Derivatives: An Overview of the Molecular Mechanisms Behind Pharmacological Activities. *Front Nutr,* 8**,** 664197.

SHARIFI-RAD, J., DEY, A., KOIRALA, N., SHAHEEN, S., EL OMARI, N., SALEHI, B., GOLOSHVILI, T., CIRONE SILVA, N. C., BOUYAHYA, A., VITALINI, S., VARONI, E. M., MARTORELL, M., ABDOLSHAHI, A., DOCEA, A. O., IRITI, M., CALINA, D., LES, F., LÓPEZ, V. & CARUNTU, C. 2021b. Cinnamomum Species: Bridging Phytochemistry Knowledge, Pharmacological Properties and Toxicological Safety for Health Benefits. *Frontiers in pharmacology,* 12**,** 600139-600139.

SHARIFI-RAD, J., QUISPE, C., BUTNARIU, M., ROTARIU, L. S., SYTAR, O., SESTITO, S., RAPPOSELLI, S., AKRAM, M., IQBAL, M., KRISHNA, A., KUMAR, N. V. A., BRAGA, S. S., CARDOSO, S. M., JAFERNIK, K., EKIERT, H., CRUZ-MARTINS, N., SZOPA, A., VILLAGRAN, M., MARDONES, L., MARTORELL, M., DOCEA, A. O. & CALINA, D. 2021c. Chitosan nanoparticles as a promising tool in nanomedicine with particular emphasis on oncological treatment. *Cancer cell international,* 21**,** 318-318.

SHARIFI-RAD, J., QUISPE, C., IMRAN, M., RAUF, A., NADEEM, M., GONDAL, T. A., AHMAD, B., ATIF, M., MUBARAK, M. S., SYTAR, O., ZHILINA, O. M., GARSIYA, E. R., SMERIGLIO, A., TROMBETTA, D., PONS, D. G., MARTORELL, M., CARDOSO, S. M., RAZIS, A. F. A., SUNUSI, U., KAMAL, R. M., ROTARIU, L. S., BUTNARIU, M., DOCEA, A. O. & CALINA, D. 2021d. Genistein: An Integrative Overview of Its Mode of Action, Pharmacological Properties, and Health Benefits. *Oxidative Medicine and Cellular Longevity,* 2021**,** 3268136.

SHARIFI-RAD, J., QUISPE, C., KUMAR, M., AKRAM, M., AMIN, M., IQBAL, M., KOIRALA, N., SYTAR, O., KREGIEL, D., NICOLA, S., ERTANI, A., VICTORIANO, M., KHOSRAVI-DEHAGHI, N., MARTORELL, M., ALSHEHRI, M. M., BUTNARIU, M., PENTEA, M., ROTARIU, L. S., CALINA, D., CRUZ-MARTINS, N. & CHO, W. C. 2022. <i>Hyssopus</i> Essential Oil: An Update of Its Phytochemistry, Biological Activities, and Safety Profile. *Oxidative Medicine and Cellular Longevity,* 2022**,** 8442734.

SHARIFI-RAD, J., QUISPE, C., PATRA, J. K., SINGH, Y. D., PANDA, M. K., DAS, G., ADETUNJI, C. O., MICHAEL, O. S., SYTAR, O., POLITO, L., ŽIVKOVIĆ, J., CRUZ-MARTINS, N., KLIMEK-SZCZYKUTOWICZ, M., EKIERT, H., CHOUDHARY, M. I., AYATOLLAHI, S. A., TYNYBEKOV, B., KOBARFARD, F., MUNTEAN, A. C., GROZEA, I., DAŞTAN, S. D., BUTNARIU, M., SZOPA, A. & CALINA, D. 2021e. Paclitaxel: Application in Modern Oncology and Nanomedicine-Based Cancer Therapy. *Oxidative Medicine and Cellular Longevity,* 2021**,** 3687700.

SINGH, B., KUMAR, A., JOSHI, P., GURU, S. K., KUMAR, S., WANI, Z. A., MAHAJAN, G., HUSSAIN, A., QAZI, A. K. & KUMAR, A. 2015. Colchicine derivatives with potent anticancer activity and reduced P-glycoprotein induction liability. *Organic & biomolecular chemistry,* 13**,** 5674-5689.

SMITH, I., HEDLEY, D., COOMBES, R. & POWLES, T. 1980. A comparison of combination chemotherapy using vindesine or vincristine with adriamycin in the treatment of advanced breast carcinoma. *Cancer treatment reviews,* 7**,** 71-73.

SUSSMAN, J. S., BROZENA, S. C., SKOP, N., KORECKA, M. & SHAW, L. M. 2004. Accidental intravenous colchicine poisoning. *Therapeutic drug monitoring,* 26**,** 688-692.

TAHER, Z. M., AGOUILLAL, F., MAROF, A. Q., DAILIN, D. J., NURJAYADI, M., RAZIF, E. N., GOMAA, S. E. & EL ENSHASY, H. A. 2019. Anticancer Molecules from Catharanthus roseus. *Indonesian Journal of Pharmacy,* 30**,** 147.

TIONG, S. H., LOOI, C. Y., ARYA, A., WONG, W. F., HAZNI, H., MUSTAFA, M. R. & AWANG, K. 2015. Vindogentianine, a hypoglycemic alkaloid from Catharanthus roseus (L.) G. Don (Apocynaceae). *Fitoterapia,* 102**,** 182-188.

TIONG, S. H., LOOI, C. Y., HAZNI, H., ARYA, A., PAYDAR, M., WONG, W. F., CHEAH, S.-C., MUSTAFA, M. R. & AWANG, K. 2013. Antidiabetic and antioxidant properties of alkaloids from Catharanthus roseus (L.) G. Don. *Molecules,* 18**,** 9770-9784.

TIWARI, D. & TIWARI, M. 2020. Vincristine: Beyond on anticancer treatment.

TOSO, R. J., JORDAN, M. A., FARRELL, K. W., MATSUMOTO, B. & WILSON, L. 1993. Kinetic stabilization of microtubule dynamic instability in vitro by vinblastine. *Biochemistry,* 32**,** 1285-1293.

TU, Y., CHENG, S., ZHANG, S., SUN, H. & XU, Z. 2013. Vincristine induces cell cycle arrest and apoptosis in SH-SY5Y human neuroblastoma cells. *International journal of molecular medicine,* 31**,** 113-119.

VIRMANI, O., SRIVASTAVA, G. & SINGH, P. 1978. Catharanthus roseus--the tropical periwinkle. *Indian Drugs*.

VISHWAKARMA, R. & PRAJAPATI, V. 2019. Drug of Vinca: Used As a Anticancer Agent”. International.

BAI, A. L. G. & AGASTIAN, P. 2013. Agrobacterium rhizogenes mediated hairy root induction for increased Colchicine content in Gloriosa superba L. *J Acad Ind Res,* 2**,** 68-73.

BINDER, B. Y., PEEBLES, C. A., SHANKS, J. V. & SAN, K. Y. 2009. The effects of UV‐B stress on the production of terpenoid indole alkaloids in Catharanthus roseus hairy roots. *Biotechnology progress,* 25**,** 861-865.

BIRAT, K., SIDDIQI, T. O., MIR, S. R., ASLAN, J., BANSAL, R., KHAN, W., DEWANGAN, R. P. & PANDA, B. P. 2021. Enhancement of vincristine under in vitro culture of Catharanthus roseus supplemented with Alternaria sesami endophytic fungal extract as a biotic elicitor. *International Microbiology***,** 1-10.

BRNÈIÆ, N., VIŠKOVIÆ, I., PERIÆ, R., ÐIRLIÆ, A., VITEZIÆ, D. & CUCULIÆ, D. 2001. Accidental plant poisoning with Colchicum autumnale: report of two cases. *Croat Med J,* 42**,** 673-675.

BUSSMANN, R. W., BATSATSASHVILI, K. & KIKVIDZE, Z. 2020. Vinca erecta Regel & Schmalhausen Apocynaceae. *In:* BATSATSASHVILI, K., KIKVIDZE, Z. & BUSSMANN, R. W. (eds.) *Ethnobotany of the Mountain Regions of Central Asia and Altai.* Cham: Springer International Publishing.

CHADBURN, H. 2014. The IUCN Red List of Threatened Species.

DARADKEH, N. Q., SHIBLI, R. A., MAKHADMEH, I. M., ALALI, F. & AL-QUDAH, T. S. Cell suspension and in vitro production of colchicine in wild colchicum hierosolymitanum Feib. The Open Conference Proceedings Journal, 2012.

DUBEY, K. K., HAQUE, S., JAWED, A., SINGH, B. P. & BEHERA, B. 2010. Construction of recombinant Escherichia coli for enhanced bioconversion of colchicine into 3-demethylated colchicine at 70 l bioreactor level. *Process Biochemistry,* 45**,** 1036-1042.

EL‐SAYED, E. S. R. 2021. Discovery of the Anticancer Drug Vinblastine from the Endophytic Alternaria alternata and Yield Improvement by Gamma Irradiation Mutagenesis. *Journal of Applied Microbiology*.

FATIMA, S., MUJIB, A. & TONK, D. 2015. NaCl amendment improves vinblastine and vincristine synthesis in Catharanthus roseus: a case of stress signalling as evidenced by antioxidant enzymes activities. *Plant Cell, Tissue and Organ Culture (PCTOC),* 121**,** 445-458.

FINNIE, J. & VAN STADEN, J. 1994. Gloriosa superba L.(Flame Lily): micropropagation and in vitro production of colchicine. *Medicinal and aromatic plants VI.* Springer.

GHOSH, B., MUKHERJEE, S., JHA, T. B. & JHA, S. 2002. Enhanced colchicine production in root cultures of Gloriosa superba by direct and indirect precursors of the biosynthetic pathway. *Biotechnology letters,* 24**,** 231-234.

GHOSH, S., GHOSH, B. & JHA, S. 2015. Role of exogenous carbohydrate and amino acid sources on biomass and colchicine production in nontransformed root cultures of Gloriosa superba. *Plant Tissue Culture and Biotechnology,* 25**,** 247-256.

GRUBBEN, G. J. H. & DENTON, O. A. 2015. Plant Resources of Tropical Africa. PROTA.

GUILLON, S., GANTET, P., THIERSAULT, M., RIDEAU, M. & TRÉMOUILLAUX-GUILLER, J. 2008. Hairy roots of Catharanthus roseus: efficient routes to monomeric indole alkaloid production. *Bioactive Molecules and Medicinal Plants.* Springer.

HAYASHI, T., YOSHIDA, K. & SANO, K. 1988. Formation of alkaloids in suspension-cultured Colchicum autumnale. *Phytochemistry,* 27**,** 1371-1374.

HUXLEY, A. 1992. The new Royal Horticultural Society dictionary of gardening.

JALEEL, C. A., GOPI, R., KISHOREKUMAR, A., MANIVANNAN, P., SANKAR, B. & PANNEERSELVAM, R. 2008. Interactive effects of triadimefon and salt stress on antioxidative status and ajmalicine accumulation in Catharanthus roseus. *Acta Physiologiae Plantarum,* 30**,** 287-292.

JALEEL, C. A., MANIVANNAN, P., SANKAR, B., KISHOREKUMAR, A., GOPI, R., SOMASUNDARAM, R. & PANNEERSELVAM, R. 2007. Induction of drought stress tolerance by ketoconazole in Catharanthus roseus is mediated by enhanced antioxidant potentials and secondary metabolite accumulation. *Colloids and surfaces B: Biointerfaces,* 60**,** 201-206.

JUNG, L. S., WINTER, S., ECKSTEIN, R. L., KRIECHBAUM, M., KARRER, G., WELK, E., ELSÄSSER, M., DONATH, T. W. & OTTE, A. 2011. Colchicum autumnale L. *Perspectives in Plant Ecology, Evolution and Systematics,* 13**,** 227-244.

KALIDASS, C., MOHAN, V. R. & DANIEL, A. 2009. Effect of auxin and cytokinin on vincristine production by callus cultures of Catharanthus roseus L.(apocynaceae). *Tropical and Subtropical Agroecosystems,* 12**,** 283-288.

KUMAR, A., PATIL, D., RAJAMOHANAN, P. R. & AHMAD, A. 2013. Isolation, purification and characterization of vinblastine and vincristine from endophytic fungus Fusarium oxysporum isolated from Catharanthus roseus. *PloS one,* 8**,** e71805.

KUMAR, C. N., JADHAV, S., TIWARI, K. & AFAQUE, Q. 2015. In vitro tuberization and colchicine content analysis of Gloriosa superba L. *Biotechnology,* 14**,** 142.

LIU, J., ZHU, J., TANG, L., WEN, W., LV, S. & YU, R. 2014. Enhancement of vindoline and vinblastine production in suspension-cultured cells of Catharanthus roseus by artemisinic acid elicitation. *World Journal of Microbiology and Biotechnology,* 30**,** 175-180.

LIU, Y., ZHAO, D.-M., ZU, Y.-G., TANG, Z.-H., ZHANG, Z.-H., JIANG, Y. & SHI, D.-Y. 2011. Effects of low light on terpenoid indole alkaloid accumulation and related biosynthetic pathway gene expression in leaves of Catharanthus roseus seedlings. *Bot Stud,* 52**,** 191-196.

MAQSOOD, M. & ABDUL, M. 2017. Yeast extract elicitation increases vinblastine and vincristine yield in protoplast derived tissues and plantlets in Catharanthus roseus. *Revista Brasileira de Farmacognosia,* 27**,** 549-556.

MEKKY, H., AL-SABAHI, J. & ABDEL-KREEM, M. 2018. Potentiating biosynthesis of the anticancer alkaloids vincristine and vinblastine in callus cultures of Catharanthus roseus. *South African Journal of Botany,* 114**,** 29-31.

MOLCHAN, O., ROMASHKO, S. & YURIN, V. 2012. L-tryptophan decarboxylase activity and tryptamine accumulation in callus cultures of Vinca minor L. *Plant Cell, Tissue and Organ Culture (PCTOC),* 108**,** 535-539.

MUKHOPADHYAY, M. J. & MUKHOPADHYAY, S. 2008. A biotechnological approach for enhancement of colchicine accumulation in Iphigenia indica Kunth. *Journal of Plant Biochemistry and Biotechnology,* 17**,** 185-188.

NGUYEN, P. A. T., KIM, J. S. & KIM, J.-H. 2015. The complete chloroplast genome of colchicine plants (Colchicum autumnale L. and Gloriosa superba L.) and its application for identifying the genus. *Planta,* 242**,** 223-237.

OSMAN, M. E., ELFEKY, S. S., EL-SOUD, K. A. & HASAN, A. M. 2007. Response of Catharanthus roseus shoots to salinity and drought in relation to vincristine alkaloid content. *Asian Journal of Plant Sciences*.

PAEIZI, M., KARIMI, F. & RAZAVI, K. 2018. Changes in medicinal alkaloids production and expression of related regulatory and biosynthetic genes in response to silver nitrate combined with methyl jasmonate in Catharanthus roseus in vitro propagated shoots. *Plant Physiology and Biochemistry,* 132**,** 623-632.

PALEM, P. P., KURIAKOSE, G. C. & JAYABASKARAN, C. 2015. An endophytic fungus, Talaromyces radicus, isolated from Catharanthus roseus, produces vincristine and vinblastine, which induce apoptotic cell death. *PloS one,* 10**,** e0144476.

PANDEY, D. K., MALIK, T., DEY, A., SINGH, J. & BANIK, R. 2014. Improved growth and colchicine concentration in Gloriosa Superba on mycorrhizal inoculation supplemented with phosphorus-fertilizer. *African Journal of Traditional, Complementary and Alternative Medicines,* 11**,** 439-446.

PARTHASARATHY, R., SHANMUGANATHAN, R. & PUGAZHENDHI, A. 2020. Vinblastine production by the endophytic fungus Curvularia verruculosa from the leaves of Catharanthus roseus and its in vitro cytotoxicity against HeLa cell line. *Analytical biochemistry,* 593**,** 113530.

PLIANKONG, P., SUKSA-ARD, P. & WANNAKRAIROJ, S. 2018. Chitosan elicitation for enhancing of vincristine and vinblastine accumulation in cell culture of Catharanthus roseus (L.) G. Don. *J Agric Sci,* 10**,** 287-293.

POULEV, A., BOMBARDELLI, E., PONZONE, C. & ZENK, M. H. 1995. Regioselective bioconversion of colchicine and thiocolchicine into their corresponding 3-demethyl derivatives. *Journal of fermentation and bioengineering,* 79**,** 33-38.

RAI, V., TANDON, P. K. & KHATOON, S. 2014. Effect of chromium on antioxidant potential of Catharanthus roseus varieties and production of their anticancer alkaloids: vincristine and vinblastine. *BioMed research international,* 2014.

SHARMA, A., MATHUR, A. K., GANPATHY, J., JOSHI, B. & PATEL, P. 2019. Effect of abiotic elicitation and pathway precursors feeding over terpenoid indole alkaloids production in multiple shoot and callus cultures of Catharanthus roseus. *Biologia,* 74**,** 543-553.

SHIBLI, R. A., DARADKAH, N. Q., MAKHADMEH, I. M. & BAGHDADI, S. H. 2010. Colchicine Production from Colchicum and the Role of in vitro Cultures: A Review. *Jordan Journal of Agricultural Sciences,* 6.

SIVAKUMAR, G. 2018. Upstream biomanufacturing of pharmaceutical colchicine. *Critical reviews in biotechnology,* 38**,** 83-92.

SIVAKUMAR, G., KRISHNAMURTHI, K. & RAJENDRAN, T. 2003. In vitro corm production in Gloriosa superba L., an Ayurvedic medicinal plant. *The Journal of Horticultural Science and Biotechnology,* 78**,** 450-453.

SIVAKUMAR, S., SIVA, G., SATHISH, S., KUMAR, G. P., VIGNESWARAN, M., VINOTH, S., KUMAR, T. S., SATHISHKUMAR, R. & JAYABALAN, N. 2019. Influence of exogenous polyamines and plant growth regulators on high frequency in vitro mass propagation of Gloriosa superba L. and its colchicine content. *Biocatalysis and Agricultural Biotechnology,* 18**,** 101030.

STEARN, W. 1978. Vinca L. *Flora of Turkey and the East Aegean Islands,* 6**,** 161-163.

VERMA, P., KHAN, S. A., MATHUR, A. K., SHANKER, K. & LAL, R. K. 2014. Regulation of vincamine biosynthesis and associated growth promoting effects through abiotic elicitation, cyclooxygenase inhibition, and precursor feeding of bioreactor grown Vinca minor hairy roots. *Applied biochemistry and biotechnology,* 173**,** 663-672.

WALDSTEIN, F. D. P. A. & KITAIBEL, P. 1802. Descriptiones et icones plantarum rariorum Hungariae.

WANG, X., PAN, Y.-J., CHANG, B.-W., HU, Y.-B., GUO, X.-R. & TANG, Z.-H. 2016. Ethylene-induced vinblastine accumulation is related to activated expression of downstream TIA pathway genes in Catharanthus roseus. *BioMed research international,* 2016.

WITT, A. & LUKE, Q. 2017. *Guide to the naturalized and invasive plants of eastern Africa*, CABI.

XU, S., REN, N., LIU, J., WU, Y. & YUAN, G. 2020. Improvement of vincamine production of endophytic fungus through inactivated protoplast fusion. *International Microbiology***,** 1-11.

YADAV, K., AGGARWAL, A. & SINGH, N. 2013. Arbuscular mycorrhizal fungi (AMF) induced acclimatization, growth enhancement and colchicine content of micropropagated Gloriosa superba L. plantlets. *Industrial Crops and Products,* 45**,** 88-93.

YOSHIDA, K., HAYASHI, T. & SANO, K. 1988. Colchicine precursors and the formation of alkaloids in suspension-cultured Colchicum autumnale. *Phytochemistry,* 27**,** 1375-1378.

ZAREV, Y., POPOVA, P., FOUBERT, K., APERS, S., VLIETINCK, A., PIETERS, L. & IONKOVA, I. 2019. Biotransformation to produce the anticancer compound colchicoside using cell suspension cultures of Astragalus vesicarius plant species. *Natural Product Communications,* 14**,** 1934578X1901400108.

ZHOU, M. & MEMELINK, J. 2016. Jasmonate-responsive transcription factors regulating plant secondary metabolism. *Biotechnology Advances,* 34**,** 441-449.
